# Versatile Magneto‐Dielectric Response of Epitaxial Thin Films of the High Entropy Oxide Perovskite Nd(Cr_0.2_Mn_0.2_Fe_0.2_Co_0.2_Ni_0.2_)O_3_

**DOI:** 10.1002/adma.72992

**Published:** 2026-04-05

**Authors:** Roxana Capu, Ryan Thompson, C. Willem Rischau, Marli R. Cantarino, Premysl Marsik, Sergey L. Bud'ko, Neven Biškup, María Varela, Yurii G. Pashkevich, Serhii M. Orel, Thomas Prokscha, Andreas Suter, Jiangtao Zhao, Ugwumsinachi Oji, Marco Bonura, Peter Bencok, Zaher Salman, Stefano Gariglio, Christian Bernhard, Subhrangsu Sarkar

**Affiliations:** ^1^ Department of Physics West University of Timisoara Timisoara Romania; ^2^ Department of Physics and Fribourg Center for Nanomaterials University of Fribourg Fribourg Switzerland; ^3^ Department of Quantum Matter Physics (DQMP) University of Geneva Geneva Switzerland; ^4^ European Synchrotron Radiation Facility France; ^5^ Ames National Laboratory and Department of Physics and Astronomy Iowa State University Ames Iowa USA; ^6^ Departamento de Física de Materiales and Instituto Pluridisciplinar Universidad Complutense de Madrid Madrid Spain; ^7^ O.O. Galkin Donetsk Institute for Physics and Engineering NAS of Ukraine Kyiv Ukraine; ^8^ PSI Center for Neutron and Muon Sciences CNM Villigen Switzerland; ^9^ Diamond Light Source Harwell Science and Innovation Campus Chilton Didcot U.K

**Keywords:** high entropy oxides, high‐k dielectrics, magneto‐dielectric coupling, magnetism, µSR, magnetostriction, perovskites

## Abstract

We report the dielectric and magnetic properties of epitaxial thin films of the high entropy oxide (HEO) perovskite Nd(Cr_0.2_Mn_0.2_Fe_0.2_Co_0.2_Ni_0.2_)O_3,_ which orders magnetically below *T*_*mag*_≈190 K. At *T* ≫ *T*_*mag*_, the dielectric response reveals a Debye‐type frequency dependence with a zero‐frequency dielectric constant of ${\varepsilon^{\prime }}_{r}$≈230–250. The dc bias voltage loops of ${\varepsilon^{\prime }}_{r}$ are reversible but exhibit three distinct peaks centred at zero and finite positive and negative voltage. We provide evidence that the zero‐bias peak is governed by the oxygen sublattice while the finite bias peaks originate from cationic dipoles. The maximal response of the latter appears to be shifted to finite bias by a static uncompensated electric field due to a vertical gradient of the oxygen content. Below *T*_*mag*_, this anomalous dielectric response is strongly suppressed, presumably by magnetostriction that counteracts and freezes the ionic displacements. These findings indicate a unique correlation between configurational entropy, dielectric response, and magnetic properties. In combination with a large dielectric strength, it enables a non‐hysteretic tuning of the dielectric response of magnetoelectronic devices with multiple parameters like temperature, electric, and magnetic field. This HEO is equally interesting for fundamental studies of competing electric and magnetic orders in strongly disordered materials.

## Graphical abstract

The magnetic high entropy oxide perovskite Nd(Cr_0.2_Mn_0.2_Fe_0.2_Co_0.2_Ni_0.2_)O_3_ exhibits a substantially large dielectric constant (*ε*_*r*_) at room temperature, which shows distinct anionic and cationic contributions in the form of zero and finite bias peaks, respectively, down to its magnetic transition temperature (*T*_*mag*_). Below *T*_*mag*_, *ε*_*r*_ sharply reduces following the non‐magnetic volume fraction, indicating coupling between magnetism and dielectric behavior.

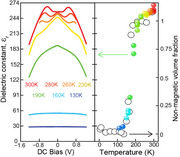

## 1 Introduction

Metal–oxide field effect transistors are the basic building blocks of modern electronic devices [1, 2, 3]. The packing density and performance of such devices rely on the properties of the dielectric layer at the gate. Hence, it has been a constant effort to find new kinds of dielectrics that have a large dielectric constant, are highly resistive, and can be readily tuned with an external electric field or other control parameters. Such dielectric thin films with large, non‐linear responses could find potential applications in standard electronic platforms such as random access memory (RAM) [4, 5, 6], AC signal generators [7], Analog‐to‐Digital Converters (ADCs) [8] and Digital‐to‐Analog Converters (DACs) [9] and microprocessors, etc. Beside standard electronics, thin film capacitors could also be used in many upcoming technological developments that include resistive random access memory (RRAM) [10], neuromorphic computing [11], memristors [12], energy storage devices [13, 14, 15], and biosensors [16].

Presently, dielectrics such as SiO_2_, HfO_2_, ZrO_2,_ and Al_2_O_3_ are the most commonly used dielectrics in the electronics industry. Their DC dielectric constants (${\varepsilon^{\prime }}_{r}$) are typically rather small, that is, in the range of 5–30 [17, 18, 19, 20, 21, 22]. Alternatively, titanate‐based perovskite ferroelectric/piezoelectric materials are being extensively studied for their potential applications [23, 24, 25]. They promise a much larger dielectric constant even at room temperature due to the relatively small size of the Ti^4+^‐ion at the B‐site (in ABO_3_ perovskite structure), which is responsible for their displacive para/ferro/flexoelectric properties. Compared to alternative candidates, like HfO_2_ [19] and ZrO_2_ [21] the perovskites have the added advantage of a strongly non‐linear characteristic of the electric field (*E*) dependence of ${\varepsilon^{\prime }}_{r}$. However, these ferroelectric materials, such as BaTiO_3_, display a large ${\varepsilon^{\prime }}_{r}$ in conjunction with a hysteretic response to an external DC electric field, that can be used for memory devices, but also makes them slow and less energy efficient because their DC response is not reversible with respect to the applied external electric field. This calls for the search of materials that have a strong and nonlinear dielectric response that is not hysteretic.

In this paper, we report on the unusual dielectric properties of a relatively new class of material, namely the magnetic high entropy oxide (HEO) perovskites. The structure of the HEO materials is stabilized by the high value of their configurational entropy that results from a random distribution of five or more cations [26] in a quasi‐equimolar ratio that is homogenous on a mesoscopic scale [27]. They surprise with their diverse and versatile properties that give rise to rich physico‐chemical phase diagrams with emergent properties like ferrimagnetism [27, 28, 29], providing new perspectives for material design and discovery.

The HEO were first synthesized and studied in the form of bulk polycrystalline powders [26, 30, 31]. Their magnetic and dielectric properties have been reported in several works. [27, 32, 33, 34, 35, 36, 37, 38, 39, 40, 41] HEO can be stabilized in different forms, including rocksalt (AB) [42], spinel (AB_2_O_4_) [43] and perovskite (ABO_3_) [44, 45]. Bulk single crystals of these materials are not yet available. However, there are several reports of the successful growth of epitaxial thin films, mostly in perovskite structure [46, 47, 48].

The magnetic properties of a perovskite class of HEO with five different magnetic ions on the B‐site thin films have been previously reported for a variety of RE(Cr_0.2_Mn_0.2_Fe_0.2_Co_0.2_Ni_0.2_)O_3_ (with RE = La [49], Tb [50], Lu [51], Dy [52], and Ho [53]). These films are typically found to exhibit an anisotropic magnetization that can be readily tuned via the strain imposed by substrates with different lattice parameters or symmetry [49]. The nature of the underlying magnetic order remains a matter of debate. Early neutron diffraction experiments indicate a coexistence of antiferromagnetic (AFM) and ferromagnetic (FM) order [48]. Subsequently, Mazza et al. [54] provided evidence that this magnetic order can be tuned by substitutional doping with Sr on the A sites—leading to finite exchange bias and antiferromagnetic spin reversal effects. Element‐sensitive X‐ray magnetic circular dichroism (XMCD) experiments have confirmed the presence of a long‐range ferromagnetic/ferrimagnetic order, albeit with a rather small magnetic moment per ion (of less than one Bohr magneton). They also indicated that the ferromagnetic moment arises from Co^2+^ and Mn^4+^ ions that deviate from the nominal 3+ valency [50].

There have also been attempts to exploit the large configurational entropy to enhance the dielectric properties of HEO perovskites containing non‐magnetic ions, in particular, Ti^4+^. The latter choice has been motivated by the emblematic dielectric perovskite BaTiO_3_ [55] and other perovskites like SrTiO_3_ [56], PbTiO_3_ [57], (Pb, Zr)TiO_3_ [58], NdMnO_3_ [59, 60], NdCrO_3_ [61, 62], NdFeO_3_ [63, 64]. They exhibit large dielectric constants (${\varepsilon^{\prime }}_{r}$) that typically involve electric dipoles which arise from a displacement of the B‐site cations toward off‐center positions within the oxygen octahedra. There have already been various attempts to increase their dielectric properties by further stoichiometric modifications. For example, Sharma and coauthors [47] have demonstrated for the case of Ba(Ti_0.2_Sn_0.2_Zr_0.2_Hf_0.2_Nb_0.2_)O_3_ thin films that a mixture of several cations on the B‐site can strongly enhance the dielectric response, as compared to single crystalline relaxor ferroelectric thin films. Such an entropy‐driven enhancement of the dielectric properties is not limited to the perovskite structure but represents a general trend that applies for several crystalline symmetries [65].

This article reports the investigation of the combined dielectric and magnetic properties of epitaxial thin films of the magnetic HEO material Nd (Cr_0.2_Mn_0.2_Fe_0.2_Co_0.2_Ni_0.2_)O_3_ (that in the following, is denoted as Nd‐HEO) in order to address their mutual interaction. We demonstrate that this system exhibits a rather strong and anomalous dielectric response that remains reproducible with repeated cycling of temperature (300–77 K) and electric field, even above a very large DC field of about ±25 MV/m. In particular, we demonstrate that above the magnetic transition temperature, *T_mag_* = 190 K, the dielectric constant is rather large and exhibits a non‐linear DC bias dependence with an unusual triple‐peak structure that sets it apart from the rest of the known dielectrics. Based on a semi‐quantitative theoretical model, we argue that this non‐trivial DC‐electric field dependence arises from distinct contributions of the anionic and cationic sublattices.

Moreover, we find that this dielectric response is strongly affected by the onset of the magnetic order below *T_mag_* where it appears to be rapidly suppressed by magnetostriction effects. This mutual coupling of the dielectric and magnetic properties provides an additional degree of freedom that can be exploited for device functionalities as well as for fundamental studies of competing orders in the presence of very strong disorder.

## 2 Experimental Results

### 2.1 Sample Preparation and Characterization

Thin films of Nd(Cr_0.2_Mn_0.2_Fe_0.2_Co_0.2_Ni_0.2_)O_3_ (Nd‐HEO) were grown by Pulsed Laser Deposition (PLD) on Nb:STO (001) substrates using a KrF excimer laser with a fluence of 1.44 mJ/cm^2^ at a frequency of 7 Hz. For the target, a polycrystalline powder with the composition Nd(Cr_0.2_Mn_0.2_Fe_0.2_Co_0.2_Ni_0.2_)O_3_ has been synthesized by mixing Nd_2_O_3_, MnO_2_, Fe_2_O_3_, Cr_2_O_3_, NiO, and Co_3_O_4_ in a stoichiometric ratio and heating it in a furnace, in a first step at 1000°C and in a second one at 1200°C, for 10 h each. The stoichiometry of the obtained powder has been confirmed by XRF (PANalytical Zetium) within an error of ± 0.01 molar ratio. Subsequently, a pellet was pressed from this powder and sintered at 1350°C to obtain a dense PLD target. The deposition was performed under an oxygen pressure of 0.3 mbar, and the substrate‐target distance was 6 cm. The deposition of a film with a thickness of 27 nm required ∼21000 pulses at 7 Hz repetition rate. The films were annealed post‐deposition first at 485°C and then at 400°C (1 h each) under an oxygen pressure of 500 mbar.

**FIGURE 1:**
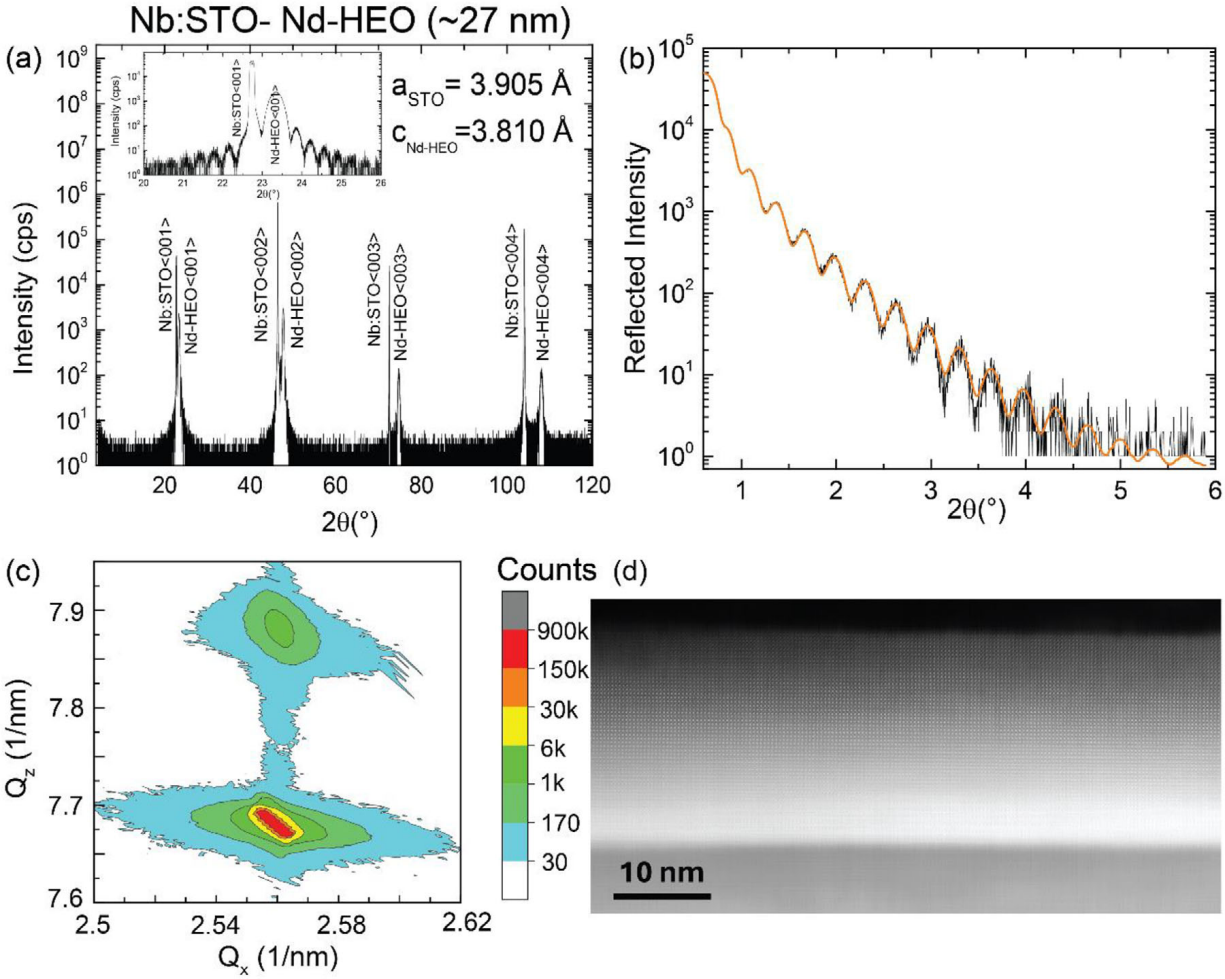
Structural characterization of the Nd‐HEO film. (a) X‐ray diffraction curve along (00L) Inset: magnified view of the 001 peaks of the film and the substrate (b) X‐ray reflectivity curve (black symbols) and best fit (orange line) that yields a film thickness of 27 nm (c) reciprocal space map (RSM) around the (013) Bragg peak (d) cross‐sectional STEM image showing an excellent crystallinity.

The structure of the Nd‐HEO thin film was analyzed by X‐ray diffraction (XRD) and X‐ray reflectivity (XRR) using a Rigaku SmartLab diffractometer with a rotating Cu‐Kα anode with a maximal power of 9 kW. Figure 1a shows the obtained <00L> curve, which exhibits several Bragg peaks of the Nd‐HEO film, that are distinct from those of the Nb:STO substrate. Also well‐resolved are thickness fringes that are superimposed on the Bragg‐peaks and characteristic of an excellent crystalline quality and a homogenous layer thickness of the thin film. The fitting of the XRR curve in Figure 1b, up to 2θ ∼ 6° using GenX [66], yields a film thickness of approx. 27 nm with rms roughness of about 0.4 nm, corresponding to an average step height of about one unit cell (1 u.c.). From the reciprocal space map (RSM) around the (0 1 3) reflection shown in Figure 1c we derived an in‐plane lattice parameter of ${\left(a/b\right)}_{HEO}=3.905\overset{˚}{A}=a_{Nb:STO}$ which suggests a lattice matched growth, without a significant relaxation of the lattice. The corresponding out‐of‐plane lattice parameter $c_{\mathrm{HEO}}=3.807\overset{˚}{A}$ is smaller than the bulk value of 3.82Å in agreement with the tensile strain state of the epitaxial thin film. Unlike the orthorhombic structure of the bulk Nd‐HEO powder [67] the RSM measured at two orthogonal azimuthal angles (013 and 103) shows no sign of an asymmetry of the in‐plane (a‐ and b‐axis) lattice parameters (within the accuracy of the instrument) and is thus indicative of a tetragonal or twinned orthorhombic structure. Figure 1d presents a cross‐sectional scanning transmission electron microscopy (STEM) high angle annular dark field (HAADF) atomic resolution image of the sample that confirms the excellent crystallinity of the thin film and also shows no signs of major lattice relaxation (in agreement with the RSM in Figure 1c). Temperature dependence of the reciprocal space map of the Nd‐HEO films was performed down to 77 K, which shows that the *c*‐axis lattice parameter reduces to 3.808 Å at 77 K from 3.902 Å at 300 K, whereas the in‐plane lattice constant remains almost constant (see Section S14 and Figure S14), exhibiting no sign of a crystallographic transition.

### 2.2 Measurement of the Dielectric Response

**FIGURE 2:**
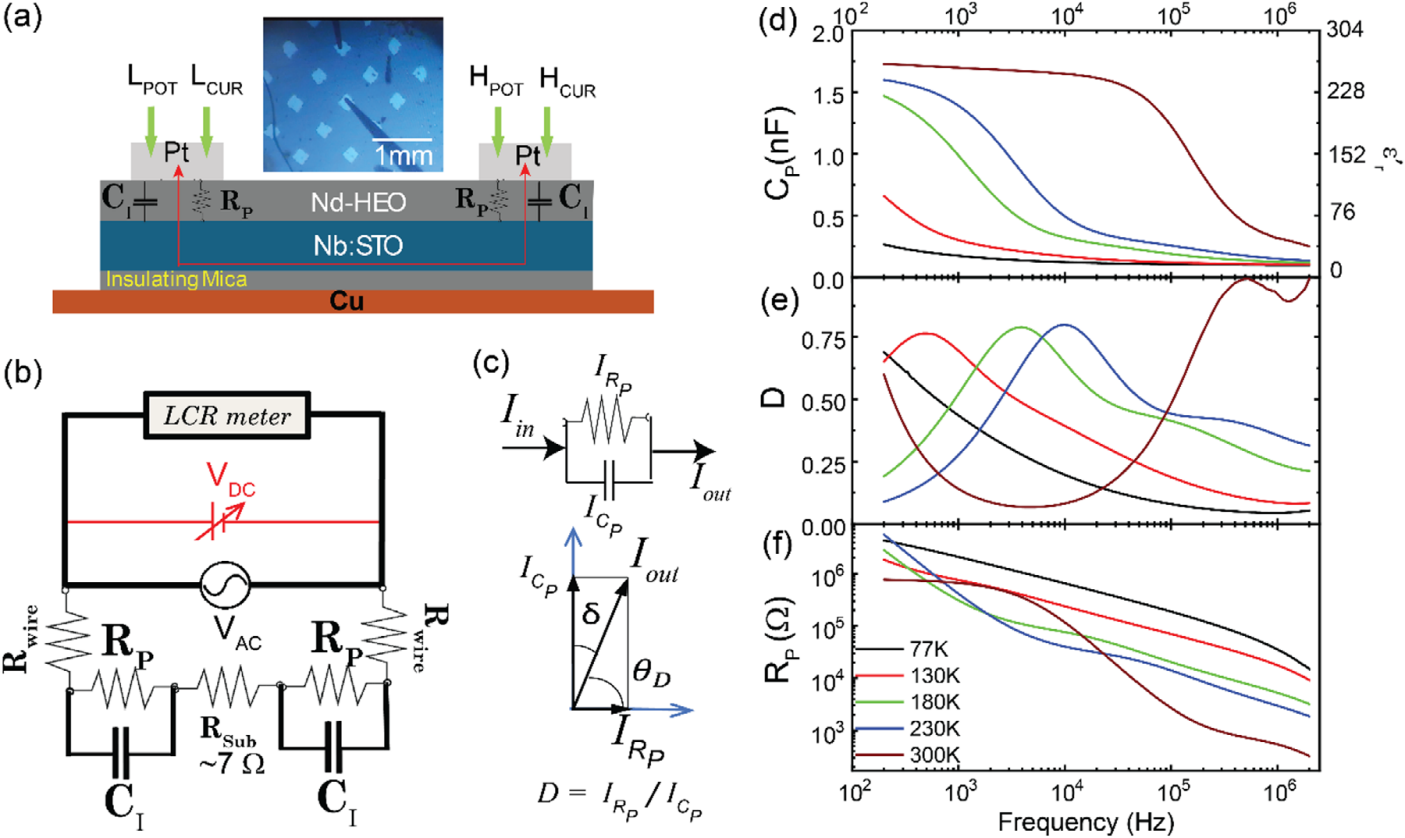
Capacitance measurements. (a) Sketch of the sample and contact assembly (b) Schematic of the electrical circuit with two parallel R‐C components that represent the current passing from the Pt contacts through the Nd‐HEO film to the Nb:STO substrate and again through the Nd‐HEO to another Pt contact. (c) Equivalent mode representation in the LCR meter. (d)‐(f) Frequency dependence at 300–77 K of the capacitance C_P,_ the dielectric function, ${\varepsilon^{\prime }}_{r}$, the loss/dissipation factor *D*, and the equivalent parallel resistance *R*_*P*_. Note that in this geometry, $C_{P}=\frac{C_{I}}{2}$, where *C*_*I*_ is the capacitance of Nd‐HEO under each Pt contact.

The out‐of‐plane dielectric response of the thin film Nd‐HEO has been measured in a configuration that uses the conducting Nb:STO substrate (Nb: 0.05 wt.%) as the bottom electrode. Figure 2a shows a sketch of the capacitors that have been produced by depositing Pt electrodes on top of the Nd‐HEO thin film. The image shows the assembly of the 200 µm x 200 µm wide Pt electrodes that have been fabricated using masks made by photo‐lithography, with a tolerance of ±5 µm. Also shown are the spring‐contacts of the probe station that have been placed on the Pt contacts in a 2‐probe geometry and connected to an Agilent E4980A LCR meter. A very thin, insulating mica sheet has been placed as a spacer between the sample and the Cu‐sample‐stage inside the cryogenic probe station, to insulate the sample electrically from the cold head while maintaining a good thermal contact.

The sample stage was cooled with liquid nitrogen from 300 K to the base temperature of 77 K. All measurements have been performed under a vacuum of< 10^−5^ mbar. The relevant response functions, for example, resistance (*R_P_*), capacitance (*C_P_*) and dissipation factor (*D*) for the equivalent circuit were acquired simultaneously at each temperature by selecting different measurement modes, such as *C_P_* − *D* and *C_P_* − *R_P_*, by sourcing a constant AC voltage of 10 mV_RMS_ between the two Pt electrodes and analyzing the complex admittance/impedance using an auto‐balancing bridge method [68]. To ensure the homogeneity of the capacitive response across the sample, the *C_P_* and *D* signals have been measured using approximately 10 combinations of contact pads. The comparison of three such combinations is shown in Figure S1.

The basic electronic circuit for this measurement geometry corresponds to a set of two parallel R‐C circuits that are connected in series via the conducting Nb:STO substrate. The resistance of the substrate (≤ 10 Ω) [69] and the probe (∼mΩ) are orders of magnitude lower than that of the sample (∼ 1 MΩ – 100 GΩ, see Figure S2). So, the measured resistance can be attributed, without any substantial error, to the thin film response.

The capacitance measured in such a controlled geometry (Figure 2b) can be directly related to the dielectric constant or the relative permittivity of the film, ${\varepsilon^{\prime }}_{r}$, according to the relationship: $C_{P}=\frac{C_{I}}{2}=\varepsilon_{0}\;\varepsilon_{r}^{\prime }\frac{A}{2d}$, where *d* is the film thickness, *A* the area of the Pt electrodes, *C_I_* is the capacitance of the individual capacitors and ε_0_ = 8.85×10^−12^ Fm^−1^ the permittivity in vacuum. The inverse factor of two accounts for the circumstance that in this serial geometry, the capacitance of the Nd‐HEO layer (*C_I_*) is measured twice.

Some additional measurements have been performed with a strongly asymmetric contact geometry, with one pole of the voltage source connected to a large pad with an area of *A*_1_ = 200 µm × 200 µm and the other to a much smaller one with *A*_2_ =  50 µm × 50 µm. For this serial circuit, the *C_P_* response is thus governed by the capacitor underneath the smaller pad. The respective equation at zero bias is $C_{P}=\varepsilon_{0}\;\varepsilon_{r}^{\prime }\frac{A1A2}{d(A1+A2)}$.

### 2.3 Frequency Dependence of *C*_*P*_ and ${\varepsilon^{\prime }}_{r}$

Figure 2d shows the frequency dependence of the measured values of *C_P_* and ${\varepsilon^{\prime }}_{r}$ of the symmetric contact geometry at several temperatures between 77 and 300 K. For the curve at 300 K, *C_P_* and ${\varepsilon^{\prime }}_{r}$ are almost constant below about 30 kHz. Notably, the extrapolated zero‐frequency value of the dielectric constant of ${\varepsilon^{\prime }}_{r}$∼ 250 at 300 K is significantly larger than for most common insulators and, in fact, quite close to that of the quantum paraelectric material SrTiO_3_ and related piezoelectric perovskites [70, 71]. The corresponding *D* vs. *f* and *R_P_* vs. *f* curves are displayed in Figure 2e,f.

**FIGURE 3:**
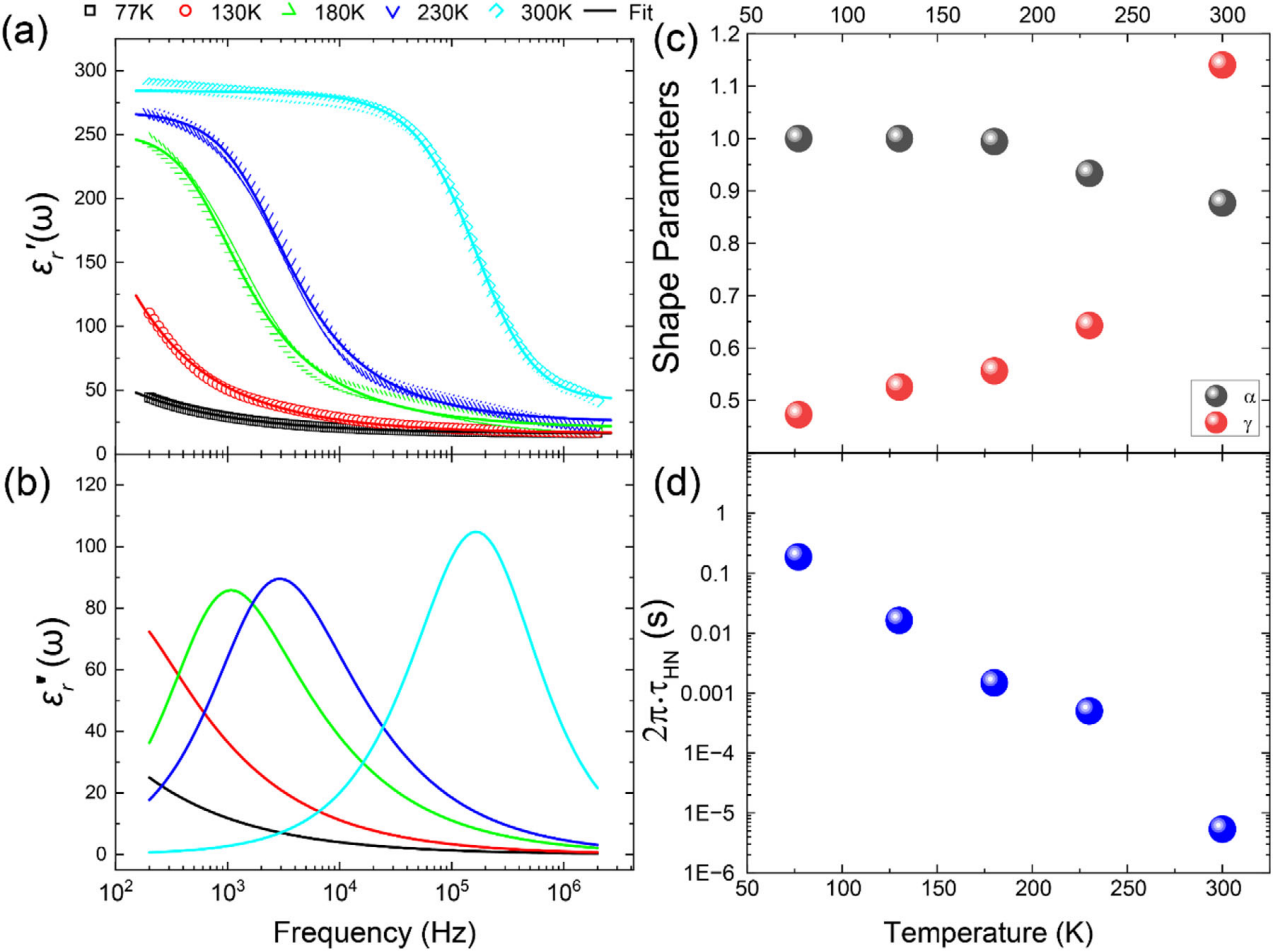
Fitting with a Havriliak–Negami relaxation model. (a) Comparison of the measured frequency dependence of ${\varepsilon^{\prime }}_{r}$ (symbols) and the best fits with the HN model (lines). (b) The imaginary part $\varepsilon_{r}^{\prime \prime }$ calculated using the parameters of the best fit shown in (a). (c) Temperature dependence of the fitted shape parameters α and γ, and (d) the relaxation time, *τ*_*HN*_.

The overall shape of the spectrum of ${\varepsilon^{\prime }}_{r}$(*f*, 300 K), which exhibits a strong decrease in the frequency range *f*: ∼ 30–500 kHz and a subsequent saturation toward higher frequencies, is characteristic of a Debye‐like response of electric dipoles. The decrease of ${\varepsilon^{\prime }}_{r}$ occurs here as the frequency of the applied AC field approaches and eventually exceeds the relaxation rate (inverse response time) of the electric dipoles. The cyan line in Figure 3a shows the best fit of the ${\varepsilon^{\prime }}_{r}$ curve with a so‐called Havriliak‐Negami function (H‐N model): [72] $\varepsilon_{r}\;\left(f\right)=\varepsilon_{\infty }+\frac{\Delta \varepsilon }{{\left(1+{\left(i2\pi f\tau_{HN}\right)}^{\alpha }\right)}^{\gamma }}$. This relaxation model describes superpositions of individual Lorentzian oscillators that results in an asymmetry (α) and broadening (γ) of a Debye‐like oscillator with relaxation time τ_*HN*_.

The corresponding complex dielectric function reads as follows [73]:

(1) $$\begin{array}{ccc} \varepsilon_{r}^{\prime }\left(f\right) & & =\varepsilon_{\infty }+\Delta \varepsilon {\left(1+2{\left(2\pi f\tau_{HN}\right)}^{\alpha }\cos\left(\frac{\pi \alpha }{2}\right)+{\left(2\pi f\tau_{HN}\right)}^{2\alpha }\right)}^{-\frac{\gamma }{2}}\\ & & \cos\left(\gamma \phi \right) \end{array}$$

(2) $$\begin{array}{ccc} \varepsilon_{r}^{\prime \prime }\;\left(f\right) & = & \Delta \varepsilon {\left(1+2{\left(2\pi f\tau_{HN}\right)}^{\alpha }\cos\left(\frac{\pi \alpha }{2}\right)+{\left(2\pi f\tau_{HN}\right)}^{2\alpha }\right)}^{-\frac{\gamma }{2}}\\ & & \sin\left(\gamma \phi \right) \end{array}$$

where $\phi =\arctan\left(\frac{{\left(2\pi f\tau_{HN}\;\right)}^{\alpha }\sin\left(\frac{\pi \alpha }{2}\right)}{1+{\left(2\pi f\tau_{HN}\right)}^{\alpha }\cos\left(\frac{\pi \alpha }{2}\right)}\right)$, Δε  = ε_*s*_  − ε_∞_ and ε_*s*_ is the static permittivity and ε_∞_ is the dielectric constant at the high frequency limit.

Figure 3a shows that the measured frequency dependence of ${\varepsilon^{\prime }}_{r}$ (symbols) can be reasonably well fitted using Equation (1) (solid lines). Figure 3b displays the corresponding frequency dependence of the imaginary part $\varepsilon_{r}^{\prime \prime }$ which has been calculated according to Equation (2) using the parameters  τ_*HN*_, ε_*s*_, ε_∞_, α, γ as obtained from the best fit to the real part ${\varepsilon^{\prime }}_{r}$. Note that the asymptotic value of ${\varepsilon^{\prime }}_{r}$ toward high frequency, that is, ε_∞_ at room temperature is similar to that measured by Infrared ellipsometery in the frequency range below the infrared active phonon modes, as shown in Figure S7 and Table S1 in Section S7. The temperature dependence of the shape parameters, α and γ, as well as the relaxation time  τ_*HN*_ are shown in Figures 3c,d, respectively.

τ_*HN*_ exhibits an almost exponential increase toward low temperature, which is also reflected in the shift of the peak in the imaginary part $\varepsilon_{r}^{\prime \prime }$. The resonance of the electric dipoles is also evident in the corresponding dissipation function *D* in Figure 2e, that represents the amplitude ratio of the in‐phase and out‐of‐phase components of the measured AC current. The latter yields the non‐dissipative (real) part of the capacitance ($C_{P}^{r}$ or ${\varepsilon^{\prime }}_{r}$) as shown in Figure 2d. The former in‐phase component contains contributions due to a finite DC conductivity (or DC resistivity *R_DC_*) and the dissipative (imaginary) part of the capacitance ($C_{P}^{i}$ or $\varepsilon_{r}^{\prime \prime }$). It is typically expressed in terms of an equivalent parallel resistance *R_P_* as shown in Figure 2f.

To demonstrate that our analysis yields a consistent description of the measured values of *R_p_* and *D* (Section S3), we used the calculated $\varepsilon_{r}^{\prime \prime }$ curve shown in Figure 3b and the measured *R_DC_* values (shown in Figure 6c; Figure S2) to estimate the frequency dependence of *R_P_* and *D* (details of the calculation can be found in Section S2). The reasonably good agreement between the calculated and the measured *D* curves shown in Figure S3 suggests that parasitic contributions, as discussed in [68], are fairly small and thus can be neglected. A comparison with measurements on standard electronic circuit elements is given in the Section S4 and Figure S4.

### 2.4 Temperature Dependence of ${\varepsilon^{\prime }}_{r}\left(f\right)$ and *D*(*f*)

**FIGURE 4:**
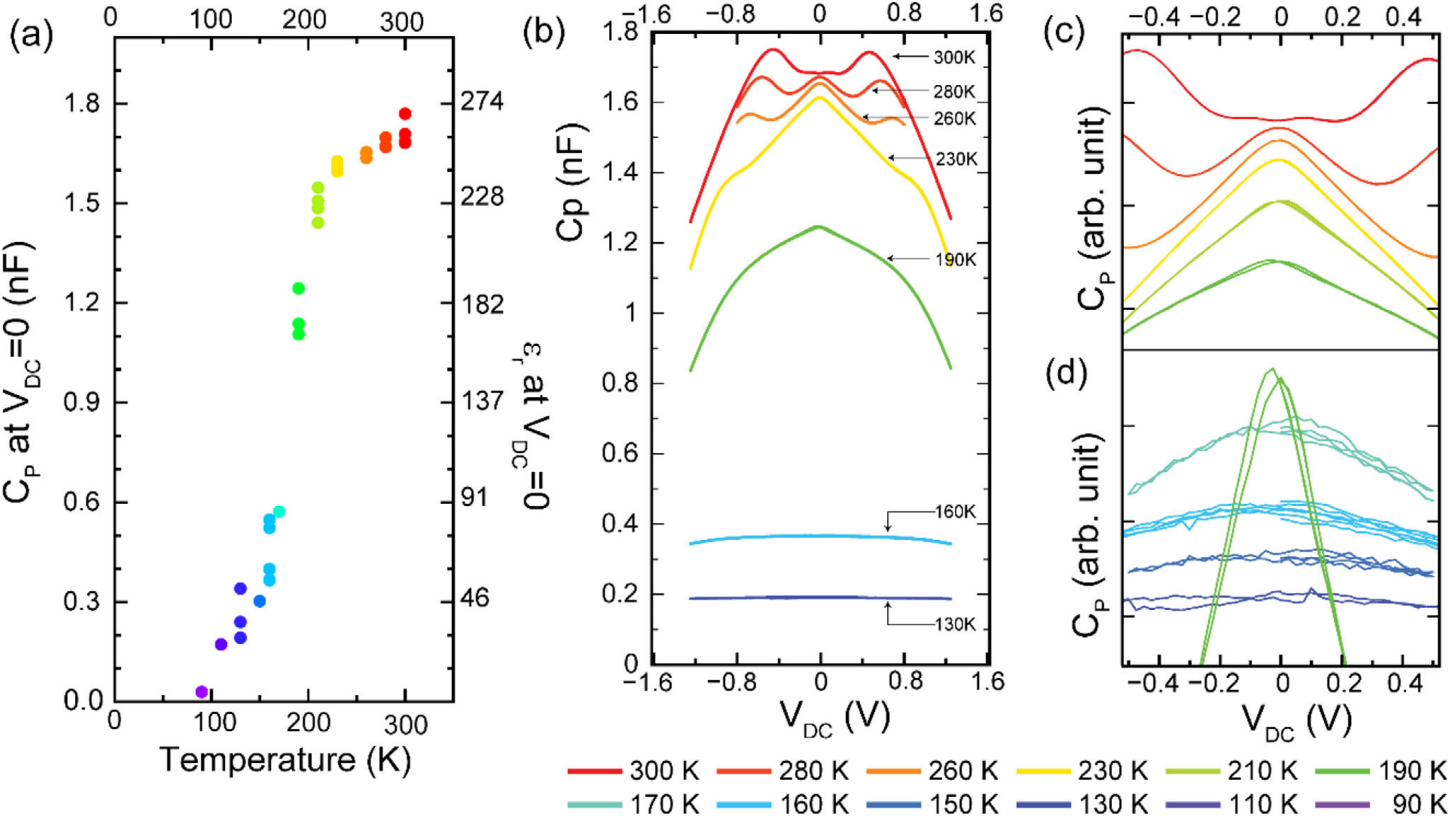
Dielectric measurements of Nd‐HEO as a function of temperature and DC bias at 1 kHz and 10 mV_RMS_ AC. (a) Dependence of the capacitance in temperature, at zero DC bias for Nd‐HEO. (b) DC‐bias (V_dc_) dependence of capacitance exhibiting the “side‐peaks”. Variation of the capacitance with electric field for the temperature range: (c) (300‐210 K) and (d) 210–90 K (10X magnified). The graphs in (c) and (d) are displayed with suitable offsets to clearly demonstrate their individual shapes. The actual values can be estimated from the color‐legends of (a).

Figure 2d,e shows the temperature evolution of the frequency curves of *C*_*P*_, $\varepsilon_{r}^{\prime }$ and *D*. They reveal a slowing down of the dynamics (relaxation rate) of the electric dipoles with decreasing temperature, which is evident from the downward shift of the edge in the $\varepsilon_{r}^{\prime }$ curve and the corresponding peak in *D* (or $\varepsilon_{r}^{\prime \prime }$). In the paramagnetic state at *T*>*T*_*mag*_∼ 190 K, the subsequent decrease of the calculated dielectric constant $\varepsilon_{r}^{\prime }$ is quite moderate. This changes rather suddenly as the magnetic order develops below *T*_*mag*_, where the loss peak in *D* eventually vanishes and correspondingly, the dielectric constant gets rapidly suppressed. This coincidence with the magnetic transition suggests that the static magnetic order gives rise to a slowing‐down and freezing of the electric dipoles. This trend is further illustrated in Figure 4a, which details the temperature dependence of the low‐frequency values (at 1 kHz) of *C*_*P*_ and $\varepsilon_{r}^{\prime }$. Note that the low temperature value of $\varepsilon_{r}^{\prime }$∼ 30 is typical for perovskite oxides without electric polar moments or instabilities [74, 75, 76, 77]. The sharp decrease of $\varepsilon_{r}^{\prime }$ below *T*_*mag*_∼ 190 K thus points toward a structural stiffening induced by an internal magnetic field, that is, a magnetostriction effect that counteracts the ionic displacements that are underlying the electric dipoles and their mobility in response to an AC electric field.

### 2.5 DC Bias Loops of *C*_*P*_ and *ε*_*r*_

Figure 4b,c show corresponding loops of *C_P_* as a function of a DC‐bias voltage that is applied between two Pt contacts (of equal area) in the form of a triangular wave alongside a 10 mV sinusoidal AC voltage with a frequency of 1 kHz. The corresponding DC electric field (*E*) can be estimated as *E*  = *V_DC_* /2*d*, with *d*  = 27 nm, neglecting non‐linear contributions that are described in Section 3.1.

The bias loops confirm the above‐described *T*‐dependent decrease of *C_P_* (and ${\varepsilon^{\prime }}_{r}$). Notably, the loops at *T* ≫ *T_mag_* reveal an unusual triple peak structure with a central peak at zero bias and two peaks at finite positive and negative bias that are symmetrically placed around the zero‐bias peak. As the temperature is reduced, the positions of the finite‐bias peaks are gradually shifted to larger *V_DC_* values and eventually they vanish below *T_mag_* (see Figure S5). Notably, the DC bias loops at *T*>*T_mag_* are fully symmetric with no sign of a hysteresis. This excludes the scenario of a ferroelectric order for which finite bias peaks can appear due to a switching of the ferroelectric domains close to the coercive field. In fact, for the dielectric response of the Nd‐HEO film a very weak hysteresis develops only at *T*<*T_mag_* where it is already strongly suppressed, and the finite bias peaks are no longer discernible.

**FIGURE 5:**
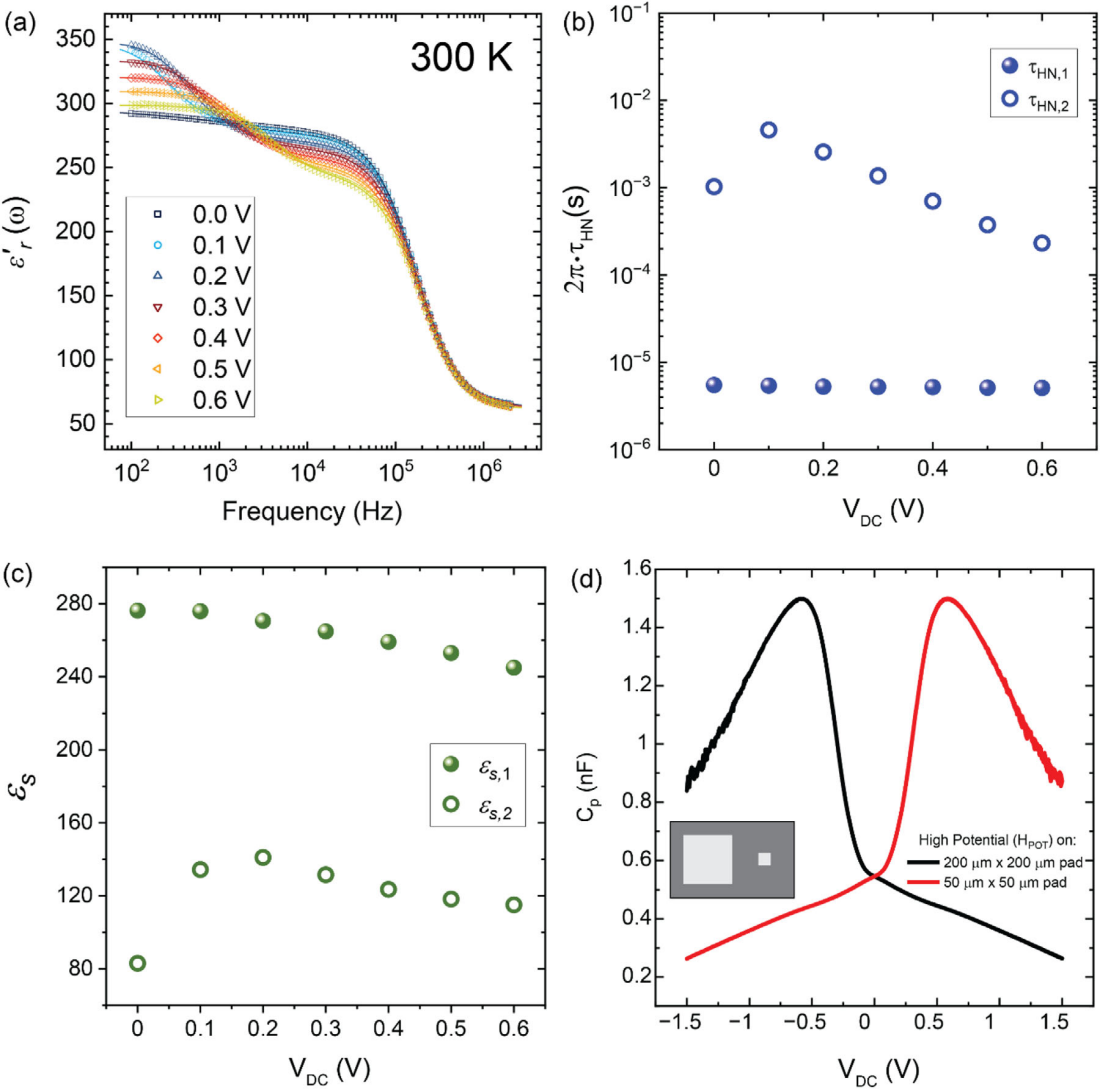
Two components of the dielectric response. (a) Frequency and DC‐bias dependence of the dielectric constant displaying a prominent second contribution under positive DC bias, (b) Time‐periods of the oscillations of faster anionic (*τ*_*HN*,1_) and the slower cationic (*τ*_*HN*,2_) oscillators calculated from the HN equation, (c) Oscillation amplitude of the anionic (*ε*_*s*,1_) and cationic (*ε*_*s*,2_) oscillators, (d) asymmetric DC bias dependence of the capacitance at 10 mV under the pad‐size asymmetry reflecting an intrinsic electric field in the volume of the thin film.

In the following, we argue that this unusual triple peak structure at T>*T_mag_* arises from two different types of paraelectric contributions to the dielectric response. Specifically, we assign the central zero‐bias peak to the anionic sublattice and the peaks at finite bias to the cationic one. The distinct origin of these two types of peaks has been confirmed by additional measurements of the frequency dependence of *C_P_* as a function of *V_DC_* at 300 K. Figure 5a shows that the experimental curves (symbols) are well reproduced by fitting with a superposition of two HN oscillators (solid lines).

The obtained best fit parameters for the relaxation times τ_*HN*,1_ and τ_*HN*,2_ and the amplitudes ε_*s*,1_ and ε_*s*,2_ of the two components are displayed in Figure 5b,c, respectively. They show that τ_*HN*,2_ is larger than τ_*HN*,1_ by about two to three orders of magnitude. Moreover, they reveal that ε_*s*,2_ is quite a bit smaller than ε_*s*,1_ and decreases toward zero bias below a broad peak around *V_DC_* = 0.2 V. Accordingly, we assign the first (second) component to the response of the anionic (cationic) subsystem. Moreover, it becomes evident that ε_*s*,2_ exhibits a maximum around *V_DC_* = 0.2 V below which it suddenly decreases. In the following, we argue that this is the signature of a static internal electric field that breaks the symmetry of the double‐well potential of the cationic dipoles and thus inhibits their response at low or zero DC bias.

The hypothesis of an internal static electric field has been confirmed by an additional measurement of *C_P_* vs. *V_DC_* at 300 K and 1 kHz for which we used contact pads with very dissimilar areas of 200 µm × 200 µm and 50 µm × 50 µm as two electrodes. The corresponding *C_P_* vs. *V_DC_* curves in Figure 5d reveal indeed a very strong asymmetry between positive and negative DC bias. The origin of the underlying static internal electric field will be discussed further below in terms of an uncompensated electric field (*E_UC_*) due to a vertical gradient of the oxygen concentration in the Nd‐HEO film.

### 2.6 Comparison of *C*_*P*_(*T*), Magnetic Volume Fraction and Second Harmonic Component

Next, we address the sudden suppression of the dielectric response below *T_mag_* and the development of a weak hysteresis that can be explained in terms of strong magneto‐dielectric coupling, for which the magnetostriction counteracts and eventually freezes the ionic displacements that govern the dielectric response.

**FIGURE 6:**
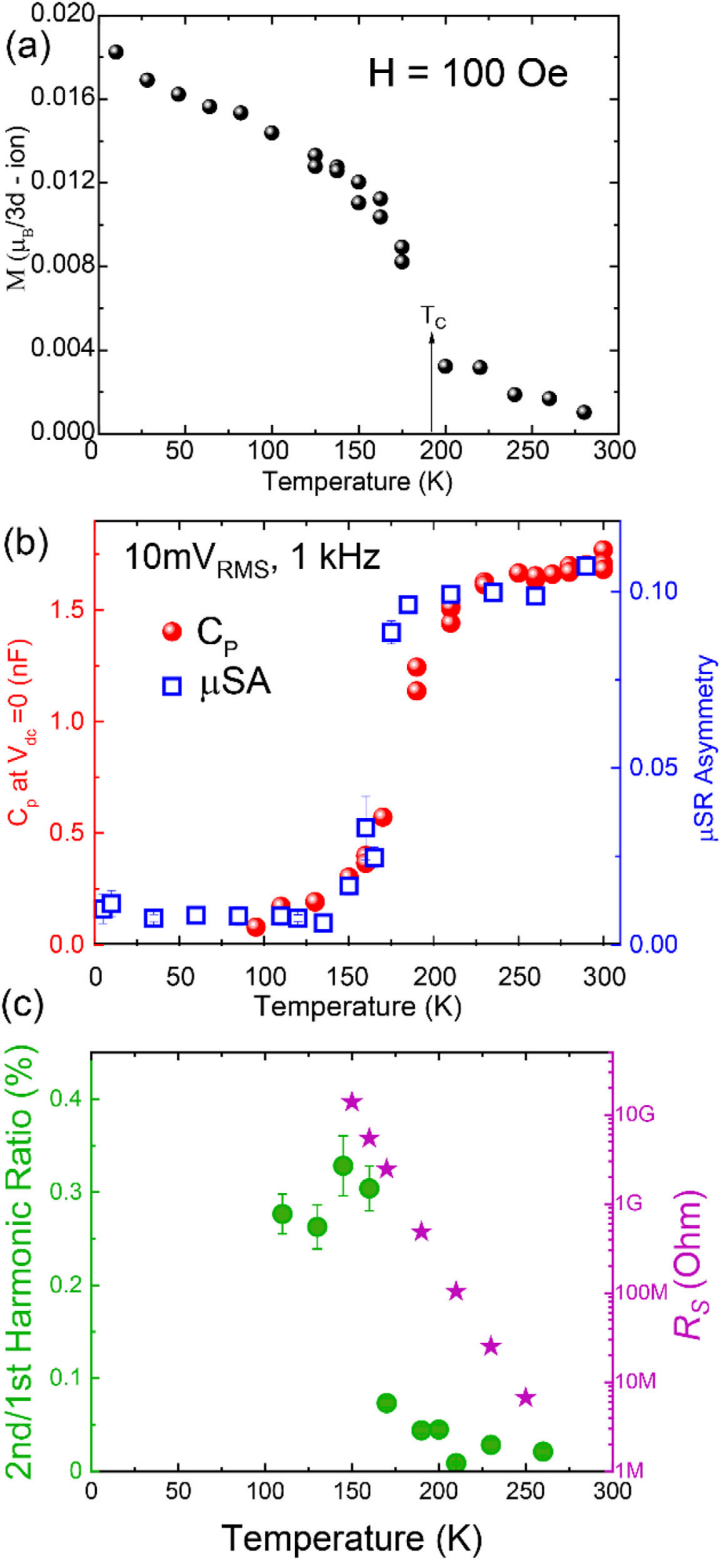
Coupling of dielectric and magnetic properties. (a) Temperature dependence of the magnetization of Nd‐HEO (in‐plane), (b) Overplot of the capacitance and µSR asymmetry with respect to temperature, and (c) the ratio of second harmonic generation (left axis) and DC resistance (right axis) with temperature.

The close relationship between the dielectric properties and the magnetic order is highlighted in Figure 6. The magnetic transition at *T_mag_* = 190 K is evident in Figure 6a, from a sudden increase of the magnetization measured with a DC SQUID for a Nd‐HEO film grown on SrTiO_3_ (001) under an in‐plane field of 100 Oe. The small value of the saturation magnetization suggests that the weak ferromagnetic moment likely arises from a canted AF or ferrimagnetic order.

The bulk‐like nature of this magnetic order has been confirmed for a corresponding 50 nm thick Nd‐HEO film grown on a LaAlO_3_ (001) substrate (which shows a similar magnetic transition like the film grown on STO, see Figure S6). Using low‐energy muon spin rotation (LEM) measurements in a weak transverse field of 100 Oe we estimate the magnetic volume fraction in the film. Here, the magnetic order gives rise to a rapid depolarization of the average muon spin in the sample that is beyond the experimental time resolution of the technique and thus seen indirectly in terms of a missing initial amplitude (asymmetry) of the oscillatory µSR signal [78]. The steep suppression of the initial µSR asymmetry in Figure 6b, which sets in below *T_mag_* and is almost complete at 130 K, thus provides unambiguous evidence for a bulk magnetic order. While the latter may only be short‐ranged and glass‐like, the LEM data establish that it persists throughout the entire sample.

For comparison, Figure 6b shows the µSR asymmetry and capacitance. It confirms a close relationship between the development of the static magnetic order and the suppression of the dielectric response. This reveals a strong magneto‐dielectric coupling which, to the best of our knowledge, has not been previously reported for a magnetic HEO.

The freezing of the electric dipoles below *T_mag_*, and the related static symmetry breaking has also been studied with a second harmonic generation (SHG) AC technique. These measurements have been performed with a SR830 lock‐in amplifier in V‐I mode. A constant AC voltage of 10 mV_RMS_, 73 Hz was sourced from the lock‐in amplifier in a 2‐probe measurement geometry across two of the Pt pads, while the first and second harmonics of the resulting current were recorded simultaneously. As displayed in Figure 6c, a sizeable second harmonic signal sets in below *T_mag_* ∼190 K and increases toward lower temperature. Note that reliable measurements of the second harmonic signal could only be performed down to about 100 K where the sample finally becomes too resistive (see Figure 6c; Figure S2). The resistance, *R_DC_*, (pink stars) as deduced from DC I‐V curves (for details see Section S2) increases steeply indeed as the temperature is reduced and reaches values in excess of hundreds of GΩ below 100 K. The common onset of the magnetic order and the SHG signal is yet another indicator for a strong correlation of the magnetic and dielectric degrees of freedom that will be further discussed later.

Following the discussion in ref. [79]. and consequent temperature dependence in *C_P_* vs. *T* (Figure 4a), we can neglect the contribution of the contacts in the observed dielectric response. See Section S8 for further details.

## 3 Semi‐Quantitative Model of Dielectric Response

In the following, we outline a minimal model that can account for the rather strong dielectric response above *T*_*mag*_ of the Nd‐HEO thin film and its characteristic evolution as a function of temperature and DC bias voltage. As was already mentioned in Section 2.6, it involves two different types of contributions to the dielectric response that originate from the anionic and cationic sublattices, respectively.

### 3.1 Dielectric Response of Anions

**FIGURE 7:**
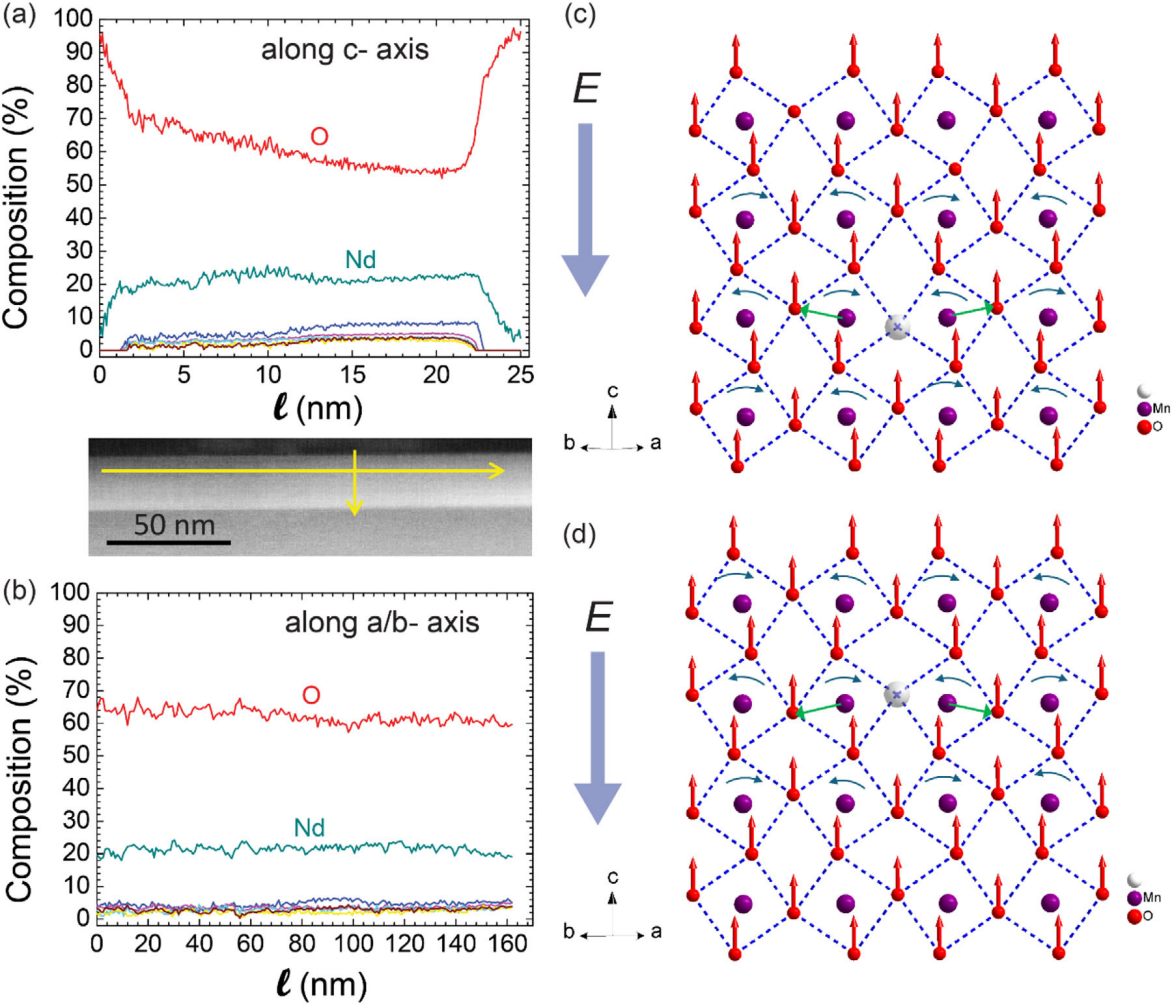
Anion contributions. STEM‐EELS map of Nd‐HEO along (a) the c‐axis from the surface toward the interface and (b) the *a*/*b* axis. The scan directions are illustrated by the yellow arrows in the middle‐pane. (c) and (d) Sketch showing the displacements of the oxygen ions in response to a uniform electric field, *E*, with an oxygen vacancy at planer Wycoff position with two possible dipole moments. The cations, oxygen ions, and vacancies are marked with purple, red, and grey spheres. In the vicinity of an oxygen vacancy, the surrounding oxygen octahedra experience a torque and undergo mutually opposite rotations, as indicated by the indigo arrows, that cause a tilting of the cation‐oxygen bonds (green arrows). The oxygen vacancies thus make the anion network softer and more responsive to an applied electric field. Oxygen vacancies at the apical position do not contribute to the change in tilting angle of the octahedra (see Section S10 and Figure S9).

For understanding the dielectric response of the anionic network, it is important to consider the role of oxygen vacancies. The distribution of the latter has been characterized with scanning transmission electron microscope‐based electron energy‐loss spectroscopy (STEM‐EELS) studies of a ∼27 nm‐thick Nd‐HEO layer. The compositional mapping derived from the STEM‐EELS spectrum images, shown in Figure 7a, reveals the presence of oxygen vacancies in Nd‐HEO, for which the concentration is maximal at the substrate‐HEO interface and gradually reduced toward the surface, where it vanishes (within the error bar). To the contrary for a scan along the lateral direction (a/b plane) the STEM‐EELS shows a homogeneous distribution of the O content.

A possible mechanism of similar oxygen vacancy formation in a high‐entropy oxide has recently been discussed in [80]. Even though the analysis considers only A‐site disorder, the discussion is still relevant for our system with B‐site disorder, since fundamentally, both systems have strained octahedrons, which are forcefully accommodated in a single crystalline lattice.

The important role of the oxygen vacancies in the dielectric response of the anionic sublattice is illustrated in Figure 7c,d. Without the oxygen vacancies, an external field causes a homogeneous overall displacement of all the oxygen ions (red arrows) that is antiparallel to *E* (large blue arrow) and yields an alternating stretching/compression of the transition metal‐oxygen (TM─O) bonds. Since this change of the cation‐anion bond length is very costly in energy the induced displacements and dipolar moments are typically rather small.

The oxygen vacancies break the charge balance of the oxygen sublattice (Figure 7d) and, in response to an applied electric field, thus give rise to a net torque on the adjacent oxygen octahedra. This torque enhances the antiphase rotations of the surrounding oxygen sublattice, which mainly involves changes of the TM─O bond angles rather than of the bond length. Accordingly, these oxygen vacancies are strongly enhancing the polarizability of the oxygen sublattice. This scenario is also compatible with the strong suppression of the dielectric response below *T*_*mag*_, where the octahedral rotations are inhibited by magnetostriction effects, which restrict the transition metal–oxygen (TM─O) bond‐angle as to maximize the superexchange interaction. The compressibility of the oxygen network is thus also expected to be very sensitive to strain effects that arise for example, from the clamping of the Nd‐HEO film to the Nb:STO substrate on which it is epitaxially grown.

The STEM‐EELS map in Figure 7a reveals a spatial gradient of the oxygen vacancy concentration along the *c*‐ axis which is expected to cause an uncompensated electric field (*E*_*UC*_) that is parallel to the surface normal of the Nd‐HEO layer.

**FIGURE 8:**
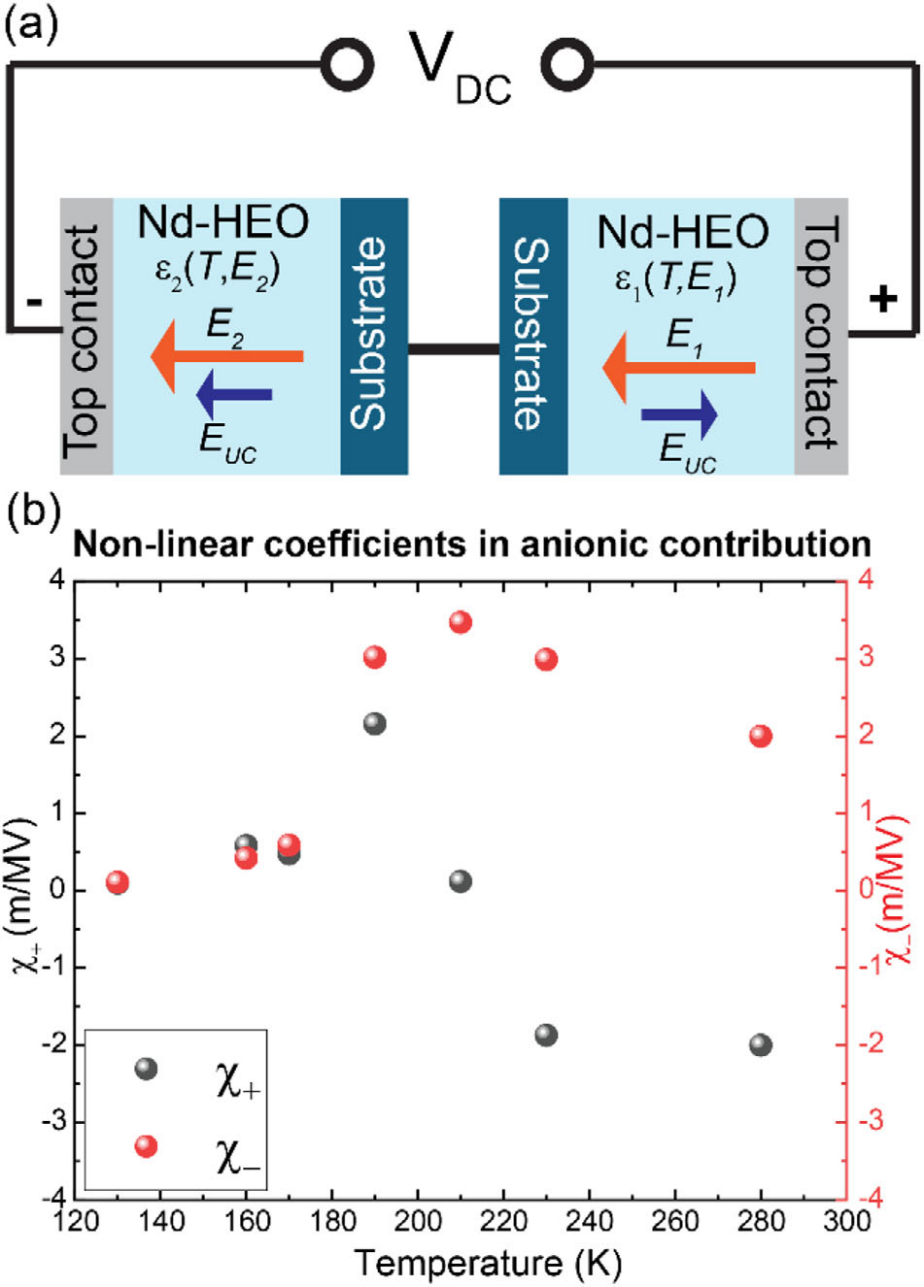
Model to describe the effect of DC electric field. (a) Simple schematic of measurement. Here, the blue bar denotes the substrate, and the grey bar denotes the platinum contacts. For any voltage sign, the electric fields (orange arrows) on both capacitors have opposite directions, either away from the substrate or toward the substrate, whereas *E*_*UC*_ (indigo arrow) always points away from the substrate. Due to nonequivalent substrate positions concerning the field direction we get inequality of dielectric constants ε_1_(T, E_1_) ≠ ε_2_(T, E_2_). (b) Calculated non‐linear coefficients of the electric field dependence of the anionic contribution in the dielectric constant following Equation (7) at different temperatures from the linear parts of Figure 4c for small values of *V*_*DC*_.

First, we discuss the fitting of the contribution of the anionic sublattice. Here, we ignore the side‐peaks at finite bias, which are assigned to the cationic response that is discussed in Section 3.2, and concentrate on the region around the central peak at zero bias. For our measurement geometry (see Figure 8a) we need to consider the combined responses of two capacitors that are connected in series.

The two internal electric fields from the combined applied DC bias and the superimposed AC voltage are pointing here in opposite directions with respect to the layer stacking order of the capacitors. As indicated by the orange arrows, the electric field is pointing toward the Nb:STO substrate in one capacitor and away from it in the other one, and vice versa if the direction of the applied voltage is reversed. Because of the symmetric nature of our electric circuit in Figure 2a, it does not directly probe the asymmetry of the dielectric response of the Nd‐HEO layers that is expected from the gradient in the density of the oxygen vacancies and the related *E_UC_*. Nevertheless, such an asymmetry has a very characteristic effect on the shape of the measured *C_P_* (*V_DC_*) curves. To show this, we express the asymmetry of the dielectric response in terms of a nonequality of the electric field dependent contributions to the dielectric function of the two capacitors:

(3) $$\varepsilon_{1}\;\left(E_{1}\right)=\varepsilon \left(0\right)+\chi_{+}E_{1};\;\varepsilon_{2}\;\left(E_{2}\right)=\varepsilon \left(0\right)-\chi_{-}E_{2}$$

Here *E*_1_, ε_1_(0) and *E*_2_, ε_2_(0) are the net electric field and the dielectric constants of the capacitors 1 and 2 at zero voltage. The coefficients χ_+_ and χ_−_ describe the nonlinearity for stretching and compression of the anionic network, respectively. The asymmetry of the dielectric response thus can be expressed in terms of the inequalities χ_+_ ≠ ‐χ_−_ and *E_1_ ≠ E_2,_* which are related according to:

(4) $$\varepsilon_{1}\left(E_{1}\right)\;E_{1}=\varepsilon_{2}\;\left(E_{2}\right)E_{2};\;\mathrm{and}\;\;V_{DC}=E_{1}\;d+E_{2}d\;$$

These equations can be rewritten as:

(5) $$\begin{array}{c} E_{1}=\frac{-\varepsilon +\chi_{-}E+\sqrt{\left(\varepsilon -\chi_{-}E\right)\left(\varepsilon +\chi_{+}E\right)}}{\chi_{+}+\chi_{-}}\;;\\ \;\;E_{2}=\frac{\varepsilon +\chi_{+}E-\sqrt{\left(\varepsilon -\chi_{-}E\right)\left(\varepsilon +\chi_{+}E\right)}}{\chi_{+}+\chi_{-}} \end{array}$$

using the notation $\varepsilon =\varepsilon \left(0\right);\;E=\frac{V_{DC}}{d}$

The capacitance of the serial configuration shown in Figure 8a amounts to:

(6) $$C_{p}\;\left(V_{DC}\right)=\frac{\varepsilon_{0}A}{d}\;\frac{\varepsilon_{1}\varepsilon_{2}}{\varepsilon_{1}+\varepsilon_{2}}$$

where *ε_0_* is the dielectric constant of vacuum, d = 27 nm and area, *A* = 4 × 10^10^ nm^2^.

At *E*  =  0, this yields $C_{p}\;\left(0\right)=1.31\times 10^{-2}\frac{\varepsilon (0)}{2}$ nF.

Finally, the voltage dependence of the measured capacitance can be written as:

(7) $$\begin{array}{ccc} & & C_{p}\left(V_{DC}\right)\\ & & =2\left[\frac{C_{0}-\frac{\chi_{+}\chi_{-}}{{\left(\chi_{+}+\chi_{-}\right)}^{2}}{\left\{\sqrt{\left(C_{0}+\kappa \chi_{+}V_{DC}\right)}-\sqrt{\left(C_{0}-\kappa \chi_{-}V_{DC}\right)}\right\}}^{2}}{C_{0}+\sqrt{\left(C_{0}-\kappa \chi_{-}V_{DC}\right)\left(C_{0}+\kappa \chi_{+}V_{DC}\right)}}\right]\\ & & \times \sqrt{\left(C_{0}-\kappa \chi_{-}V_{DC}\right)\left(C_{0}+\kappa \chi_{+}V_{DC}\right)} \end{array}$$

with the numerical dimensional coefficient κ = 0.2426 nF.m^−1^ (required to convert dimensionless ε to the units of capacitance in our measurement geometry), the non‐linear coefficients  χ_+_ and χ_−_ expressed in units of (m.MV^−1^), and *C*_0_ is the experimental value of the capacitance (in nF) at *V_DC_* =  0.

Figure 8b shows the result of fitting the experimental curves of *C_p_* (*V_DC_*) with Equation (7) at temperature from 130–280 K and in a DC bias range that is unaffected by the side peaks. Figure S8 in Section S9 highlights the overall good agreement between the experimental data and the best fit for the example of the *C_p_* (*V_DC_*) curve at 130K and 190 K. Figure 8b details the temperature dependence of the best fit parameters for the coefficients χ_+_(*T*) and χ_−_(*T*).

For a better understanding of the obtained results, in particular for the observed change of shape of the *C_p_* vs. *V_DC_* curve from 130 K, where it is almost an inverted parabola, to 190 K and above, where it exhibits a pronounced cusp at zero bias, we consider the following two limiting cases.

- The nonlinear part of the dielectric response of the anionic network is maximally asymmetric with respect to the direction of the electric field, that is, χ_+_(*T*)  = χ_−_(*T*)  = χ_*as*_(*T*). Here we derive from Equation (7) the expressions:
(8) $$C_{p}\;\left(T,V_{DC}\right)=\sqrt{C_{T0}^{2}-\kappa^{2}\chi_{as}^{2}{V_{DC}}^{2}}\;$$
(9) $$\begin{array}{c} E_{1}=\frac{-\varepsilon +\chi_{as}E+\sqrt{\varepsilon^{2}-\chi_{as}^{2}E^{2}}}{2\chi_{as}}\;;\;\\ E_{2}=\frac{\varepsilon +\chi_{as}E-\sqrt{\varepsilon^{2}-\chi_{as}^{2}E^{2}}}{2\chi_{as}} \end{array}$$
(10) $$\begin{array}{c} \varepsilon_{1}=\frac{\varepsilon +\chi_{as}E+\sqrt{\varepsilon^{2}-\chi_{as}^{2}E^{2}}}{2}\;;\;\\ \;\varepsilon_{2}=\frac{\varepsilon -\chi_{as}E+\sqrt{\varepsilon^{2}-\chi_{as}^{2}E^{2}}}{2} \end{array}$$

According to Equation (8), the *C_p_* (*T*,*V_DC_*) curve does not exbibit a cusp at *V_DC_* =  0, rendering it differentiable. Moreover, Equation (10) shows that the dielectric constants acquire an electric‐field induced asymmetry that enables second harmonic generation (SHG) in response to an AC voltage. Both features agree well with the data at T<*T_mag_* where the *C_p_* (*T*,*V_DC_*) curves do not show a cusp feature at the origin (see Figure 4c), and where a clear SHG signal is observed, as shown in Figure 6c.

- The nonlinear response of the anionic network is assumed to be fully symmetric with respect to the orientation of the electric field versus the layer stacking sequence of the capacitor with − χ_+_(*T*)  = χ_−_(*T*)  = χ_*s*_(*T*) > 0. According to Equation (7), this yields:
(11) $$C_{p}\;\left(T,V_{DC}\right)=C_{T0}\;-\frac{\kappa \chi_{s}\left(T\right)\left|V_{DC}\right|}{2}$$
(12) $$\begin{array}{c} E_{1}=E_{2}=\frac{E}{2}=\frac{V_{DC}}{2d}\;;\;\varepsilon_{1}\;\left(T,V_{DC}\right)=\varepsilon_{1}\;\left(T,V_{DC}\right)\\ =\varepsilon \left(T,0\right)-\frac{\chi_{s}\left(T\right)E}{2} \end{array}$$

Here the *C_p_* (*T*,*V_DC_*) curves exhibit a cusp around *V_DC_* =  0 (non‐differentiable) that arises from the term linear in *V_DC_* in Equation (11). Moreover, there can be no SHG signal since Equation (12) yields equal dielectric constants in both capacitors. The experimental data in Figures 4 and 6 and the fitted nonlinear coefficients shown in Figure 8b indicate that the anionic subsystem approaches this limit above *T_mag_*, for example, at *T* ≥ 230 K.

Such a crossover from a strongly (or even fully) asymmetric non‐linear part of the dielectric constant below *T_mag_* (scenario 1) to an (almost) fully symmetric one above *T_mag_* (scenario 2) can be understood in terms of the clamping of the epitaxial Nd‐HEO layer to the Nb:STO substrate that depends on the strength of the polarizability and the related compressibility of the oxygen sublattice. Below *T_mag_*, where the polarizability is strongly reduced by the magnetostriction, this clamping is only gradually released along the vertical direction and thus give rise to a strong asymmetry of the field‐induced non‐linear part of the dielectric function. To the contrary, well above *T_mag_* the Nd‐HEO lattice is softer and relaxes much faster. The non‐linear part of the dielectric function thus becomes almost symmetric, since it hardly depends anymore on the direction with respect to the Nb:STO substrate in which the oxygen ions are displaced. The equations for the temperature and voltage dependence of ε (*T*, *V*) are detailed in Section S10.

To stress further, the above‐described scenario is also consistent with the circumstance that a finite SHG signal is observed only below *T*_*mag*_. With the experimental setup shown in Figure 2b, a SHG can only be detected if the two capacitors connected in series have different non‐linear responses. The finite SHG signal below *T*_*mag*_ is thus indicative of a rather strong asymmetry of the dielectric response concerning the orientation of the electric field with respect to the stacking order of the Nd‐HEO layer and the Nb‐STO substrate.

### 3.2 Dielectric Response of Cations

Compounds like SrTiO_3_ [81] or BaTiO_3_ [82] with Ti^4+^ ions are well known for their strong dielectric response of electric dipoles that arise from the off‐center displacement of the Ti cations toward the neighboring oxygen ions. Such a spontaneous displacement requires that the Ti─O bond length exceeds a critical value above which the energy gain due to the shortened Ti─O bonds outweighs the energy loss of the Ti─O bonds that are expanded. The occupation of these off‐center positions and the mutual transitions between them can be described in terms of a double well potential with two minima that are separated by an energy barrier. The latter barriers, which depend on the cationic species, their valency, and the temperature, need to be overcome for the cationic dipoles to be reversed, for example, in response to an AC electric field. For several titanates like BaTiO_3_ [82] or doped SrTiO_3_ [83, 84, 85, 86, 87, 88, 89]_,_ such a paraelectric behavior of the cationic dipoles is only observed at rather higher temperatures, whereas below a certain transition temperature, dipoles tend to develop a long‐range ordered, ferroelectric state.

For the present Nd‐HEO system, we consider that some of the TM‐cations may also be prone to off‐center displacements that give rise to cationic dipoles. However, due to the random distribution of the TM cations, it is not expected that these rather dilute and randomly distributed cationic dipoles develop a long‐range order. The assumption of displacement ‐induced cationic dipoles has been supported by calculations of the related change of the electronic ground state energy using a modified crystal field theory [90, 91, 92] (details can be found in Section S12). For this analysis, it is crucial to determine the valence states of the different TM cations. For the individual parent compounds with a single TM cation, that is, for Nd*M*O_3_ with *M* = Ni [93], Fe [94], Cr [95], Mn [96], or Co [97], the cations are typically constrained to the +3‐valence state. For some magnetic Nd‐HEO materials, however, it has been reported from X‐ray absorption spectroscopy (XAS) measurements that the TM cations can acquire oxidation states different from the +3 state of the parent compounds [51, 52, 53]. We have therefore performed XAS measurements at the L_2,3_ edges of Ni, Fe, Cr, Mn, and Co, to investigate the TM valence states for our Nd‐HEO material. From the comparison of the measured spectra with those obtained from model calculations (details are given in the methods and Section S12), we have obtained evidence for the following distribution of oxidation states: Ni^2+^, Fe^3+^, Cr^3+^, Mn^4+^, Co^2+^ (52%), and Co^3+^ (48%).

**FIGURE 9:**
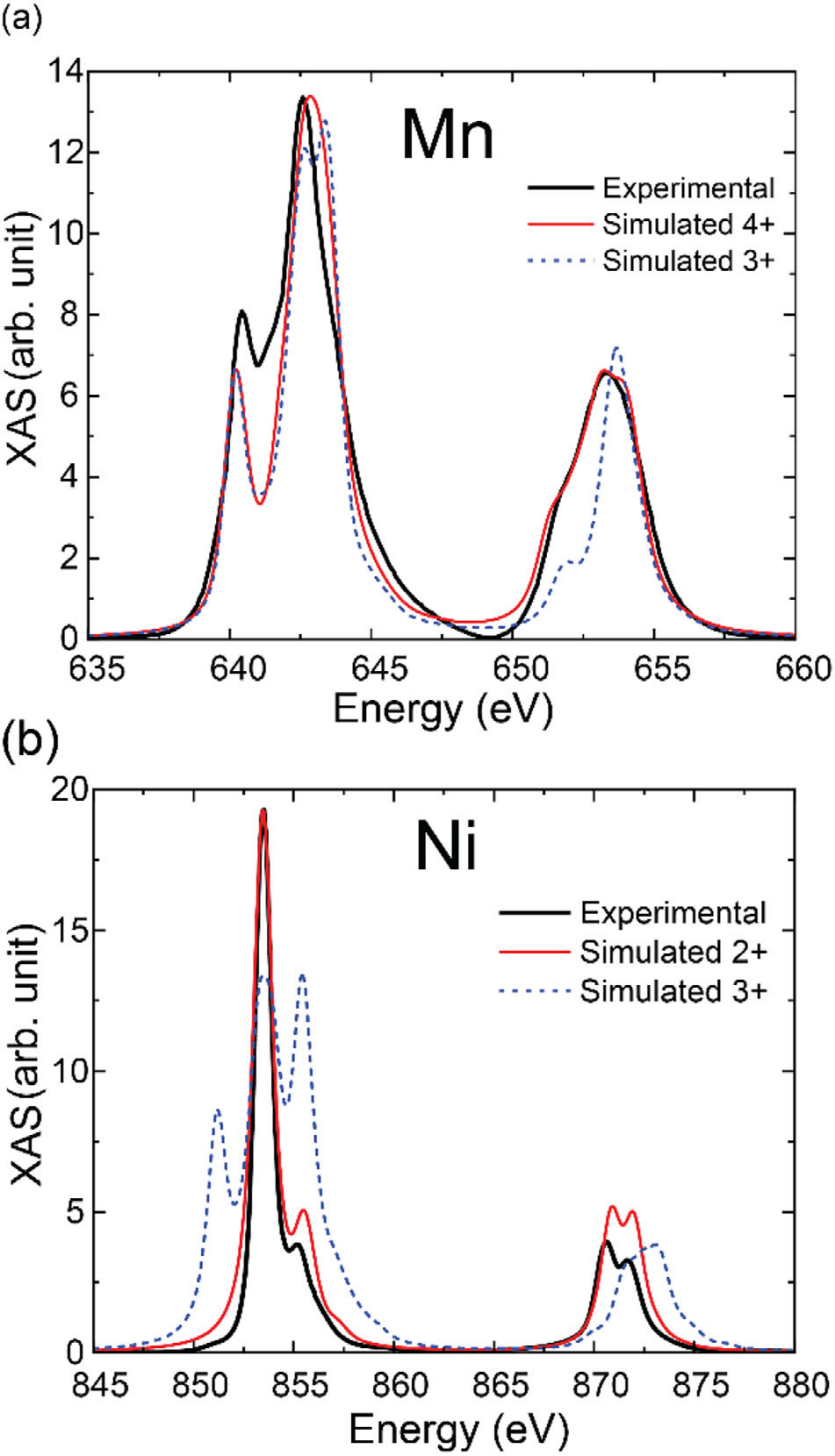
Electronic states of the cations. X‐ray Absorption Spectra for Nd‐HEO around the L_3_ and L_2_ edges of (a) Mn and (b) Ni at 10 K. The simulated spectra are also displayed in the same plots.

Figure 9a,b show a comparison of the experimental and simulated absorption spectra for the example of the Mn and Ni cations, respectively, which exhibit the largest deviations from the +3 valence state of the parent compounds and are therefore most important for the following discussion. Details of the simulation and related parameters can be found in Section S12 and Figure S14 and Table S2.

**FIGURE 10:**
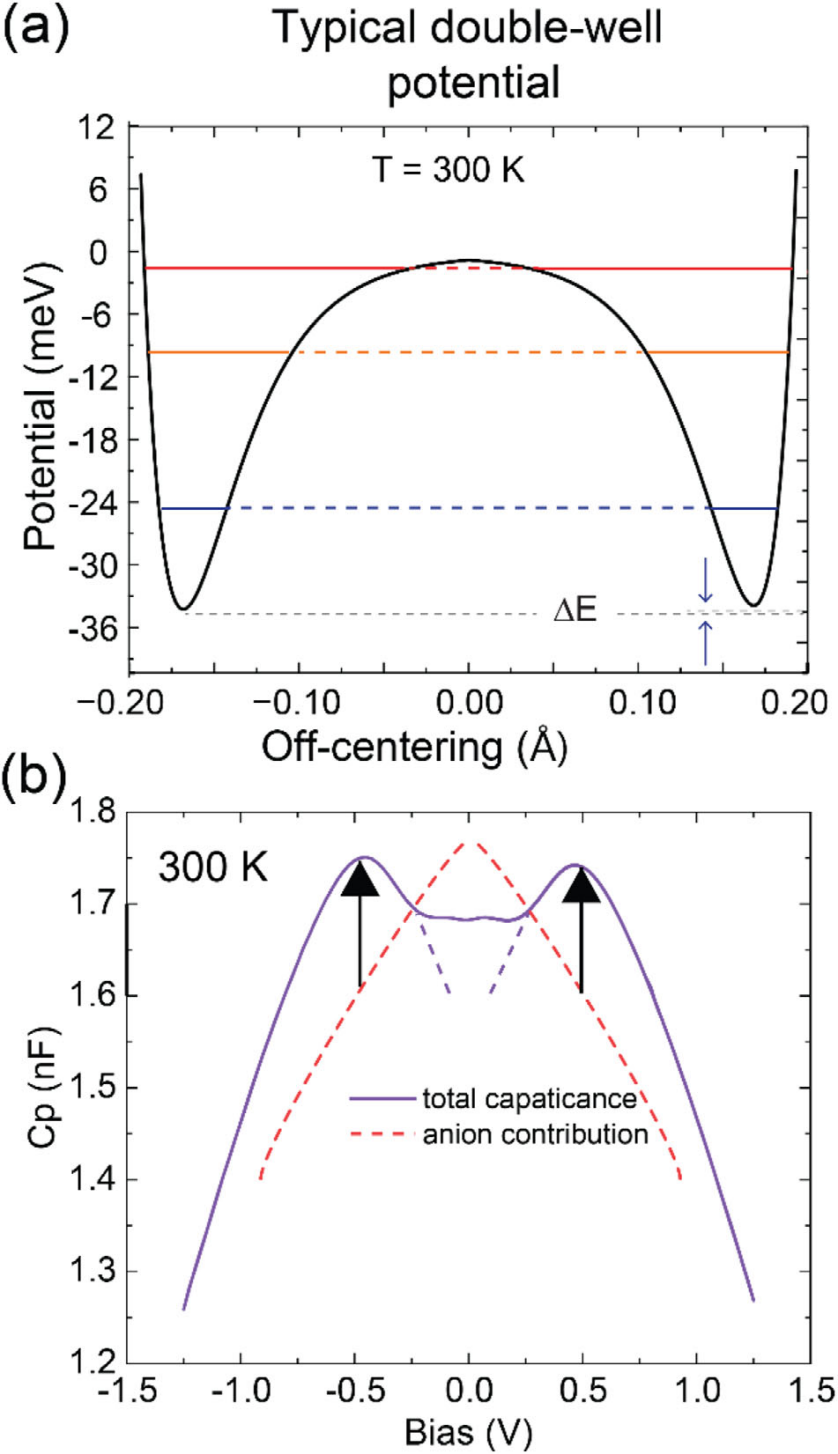
Cation contributions. (a) The calculated double‐well potential for Mn^4+^ ions under *E_UC_* exhibiting 3 available vibrational states at 300 K. The population (schematic) of the ions in the two different wells is unequal (ΔE) due to the applied field as well as *E_UC_* and transition can be activated by an application of an external electric field at the low temperature. (b) The scheme of cationic contributions on the background of the fitted anionic contributions at 300 K.

Notably, the valence state of the TM cations has a large effect on their so‐called Shannon‐radii, which determines the preferred length of the bonds with their neighboring oxygen ions. The Shannon‐radius of a +4 cation is strongly decreased, and that of a +2 cation is strongly increased, as compared to that of the corresponding +3 cations [98]. It is therefore natural to assume that its predominantly the Mn^4+^ ions that are undergoing the off‐center displacements which give rise to cationic dipoles. This assumption has been confirmed with a detailed analysis as described in Section S12, which is based on the cationic valence states obtained from the XAS data (as listed above), the crystallographic structure of the orthorhombic NdMnO_3_ parent compound [99], and the lattice parameters of the Nd‐HEO film. This analysis confirms that the cationic dipoles originate predominantly from the Mn^4+^ ions. Moreover, it reveals that the energy barrier of the double‐well potential of the Mn^4+^ ions amounts to approximately 30 meV, a value similar to the thermal energy at room temperature (Figure 10a). This analysis is consistent with our experimental finding that the cationic dielectric response is considerably weaker than that of the oxygen sublattice. In particular, for the DC bias curves around room temperature in Figure 4b we estimate, according to the schematics shown in Figure 10b, that the cationic contribution to the capacitive response amounts to less than 10% of that of the oxygen sublattice.

A second important feature of the cationic response involves the internal electric field *E_UC_* that arises from a vertical gradient of the concentration of oxygen vacancies, as has already been discussed in Section 3.1. This static field lifts the degeneracy of the local minima of the double‐well potential of the Mn^4+^ cations, which thus becomes asymmetric (Figure 10a). This asymmetric potential tends to trap the cations in the lower potential minimum, and this prohibits a reversible hoping of the Mn^4+^ cations and reduces the cationic contribution to the dielectric response. The cationic dielectric response can however be resurrected by applying an external electric field that counteracts *E_UC_* and eventually restores a symmetric double‐well potential of the Mn^4+^ cations. A further increase of the applied electric field once more introduces an asymmetry to the cationic double‐well potential, which is inverted compared to that at zero (or small) applied electric field. The interplay between *E_UC_* and the applied electric field (or DC bias voltage) thus provides a natural explanation for the finite bias peaks of the *C_P_* versus *V_DC_* curves in Figure 4b. The circumstance that the finite bias peaks in Figure 4b occur equally on the positive and negative sides of the bias voltage loops is owed to the symmetric measurement geometry (Figure 2a). Likewise, for the measurement with the strongly asymmetric contact pad geometry in Figure 5d, for which the capacitor underneath the much smaller contact dominates the serial *C_P_* signal, the asymmetry due to *E_UC_* is evident from the sharp and unipolar maximum of the *C_P_* versus *V_DC_* curve. Corresponding details have been provided in Section S10B, where the configuration has been figuratively demonstrated in Figure S10 and the estimate of the effective electric field sensed by the pads have been presented in Figure S11.

An estimate of the magnitude of *E_UC_* is complicated by the non‐linear electric‐field dependence of the anionic contribution to the dielectric response that evolves according to Equation 12. As is explained in Section S11, we can only provide an estimate of a lower bound of *E_UC_* of about 2 MV.m^−1^. As examples, two types of off‐centering directions for Mn4+ ions in the oxygen octahedron cage and the corresponding variation of the Mn^4+^ adiabatic potential have been demonstrated in Figures S12 and S13.

At last, we remark that the dielectric response of the anionic sublattice is only weakly affected by the internal field *E*_*UC*_. The relevant anti‐phase rotations of the octahedra with oxygen vacancies can be described in terms of a single minimum that lacks an activation barrier. Accordingly, the anionic dielectric response always exhibits a maximum at zero DC bias. Notably, a hopping of the oxygen vacancies between different octahedral units, which has been proposed to account for the relaxor‐type response of certain perovskite oxides [100], would give rise to finite bias peaks, albeit at a rather high electric field that is well beyond the range of our study. Hence, such a contribution from the hopping of oxygen ions (or vacancies) has not been considered in our analysis.

## 4 Discussion

We have investigated the dielectric and magnetic properties of a thin film of a magnetic high entropy oxide (Nd‐HEO) perovskite that contains five different magnetic transition metal ions and exhibits a static magnetic order below *T_mag_* = 190 K. The dielectric function of the Nd‐HEO layer has been determined as a function of frequency, temperature and applied DC‐electric field, by measuring the capacitance of a serial circuit of inversely stacked Pt/Nd‐HEO/Nb:STO and Nb:STO/Nd‐HEO/Pt capacitor structures. The development of a bulk magnetic order below *T_mag_* = 190 K has been established with DC magnetization and low‐energy muon‐spin‐rotation (LEM) measurements.

At *T*>>*T_mag_*, we observed a rather strong dielectric response with a Debye‐type frequency dependence and a sizeable zero‐frequency constant of ${\varepsilon^{\prime }}_{r}$ ∼ 230–250 similar to previous reports on epitaxial thin films of non‐magnetic HEO perovskites [47]. This aligns with the observation that the disorder due to randomly distributed cations helps to soften the oxygen sublattice and thus to increase its polarizability [32, 33, 34, 35, 36, 37, 38, 39, 40, 41, 47, 101]. For our magnetic Nd‐HEO, however, we find that the static magnetic order gives rise to a massive suppression of the dielectric response below *T_mag_* = 190 K. This indicates a strong magneto‐dielectric coupling that most likely involves magnetostriction effects that compete with the lattice distortions/displacements underlying the dielectric response.

Another key observation concerns the unusual shape of the measured capacitance curves under DC bias, which in the paramagnetic state at *T* > *T_mag_* exhibit a characteristic triple peak structure. In addition to a central peak at zero bias, the *C_P_* (*V_DC_*) curves reveal two finite bias peaks that are symmetrically placed around the zero‐bias peak on the negative and positive field branches. Notably, at *T* ≫ *T_mag_* these *C_P_* (*V_DC_*) curves are fully reversible with no sign of a hysteretic behaviour. The finite bias peaks thus do not arise from a ferroic electric order. A weak hysteresis of the *C_P_* (*V_DC_*) loops is only seen below *T_mag_*, where it is most likely caused by the coupling to the ferroic magnetic order.

The above‐described observations have been explained in terms of a semi‐quantitative model that contains distinct contributions to the dielectric response from the anionic and the cationic sublattices. In addition, this minimal model considers an internal electric field that is pointing along the surface normal of the Nd‐HEO layer and arises from an uncompensated electric field due to a vertical gradient in the oxygen vacancy concentration that is evident from the STEM‐EELS data. Additional contributions to an asymmetric dielectric response, with respect to the applied electric field, arise from the clamping of the Nd‐HEO thin film to the Nb:STO substrate on which it is epitaxially grown.

Note that the majority of our capacitive measurements have been performed using a serial circuit with equally sized Pt contact pads, as sketched in Figure 2a. For such a geometry the measured *C_P_* (*V_DC_*) loops do not directly reflect the asymmetry of the dielectric response function, that is, the *C_P_* (*V_DC_*) loops are always symmetric with respect to the sign of the applied electric field. Nevertheless, we have shown that the intrinsic *E_UC_* and the asymmetric dielectric response give rise to characteristic features of the *C_P_* (*V_DC_*) loops, like the finite bias peaks and particular shapes of the zero‐bias peak. Moreover, we have performed additional proof of principle *C_P_* (*V_DC_*) measurements using a modified contact geometry with largely different sizes of the two Pt contacts of 50 µm x 50 µm vs. 200 µm x 200 µm. Here, the capacitive response of the serial circuit is dominated by the smaller junction and an asymmetric dielectric response function of the Nd‐HEO layer thus gives rise to a corresponding asymmetry of the measured *C_P_* (*V_DC_*) curves, as is evident from Figure 8b.

Our minimal model assumes that the largest contribution to the overall dielectric response arises from the oxygen sublattice. Its large polarizability is due to a rather high concentration of oxygen vacancies that breaks the charge balance of the surrounding oxygen octahedra. In response to an external electric field, this charge imbalance gives rise to a torque that enhances the tilting of the oxygen octahedra. The soft oxygen sublattice thus governs the overall dielectric response and gives rise to a pronounced zero‐bias peak in the *C_P_* (*V_DC_*) curves. The latter has been shown to undergo a characteristic shape change as a function of temperature. At *T* ≫ *T_mag_* the zero‐bias peak is a sharp, cusp‐like feature whereas toward *T_mag_* it develops a strongly rounded, almost inverse parabolic shape. We have outlined that this reflects a corresponding change of the symmetry of the non‐linear dielectric response of the oxygen sublattice. As shown in Figure 8b, the latter is nearly symmetric with respect to the external electric field at *T* ≫ *T_mag_* whereas it becomes strongly asymmetric upon approaching *T_mag_*. This asymmetry has been explained in terms of a clamping of the epitaxially grown Nd‐HEO film to the Nb:STO substrate which renders the polarizability of the oxygen sublattice asymmetric. Above (below) *T_mag_*, where the oxygen sublattice is very (hardly) flexible, this clamping effect is confined to (still felt far away from) the Nb:STO/HEO interface.

The weaker cationic response (<10%) accounts for the finite bias peaks at *T* ≫ *T*_*mag*_ that occur on the positive and negative branches of the *C*_*P*_(*V*_*DC*_) curves. It involves electric dipoles that originate from an off‐centre displacement of some of the TM cations within their octahedral units. From XAS measurements of the oxidation states of the various TM cations and subsequent estimates of the Shannon radii of the ions, we deduced that the Mn^4+^ ions are the prime candidates to undergo such off‐center displacements. The hopping of the Mn^4+^ ions between the various off‐center positions within the octahedral units in response to an electric field is typically described by a double‐well potential. For the barrier height we obtain an estimate of about 30 meV which agrees well the observation of a gradual enhancement of the finite bias peaks as temperature increases up to room temperature. For a symmetric double‐well potential with degenerate local minima, however, the maximal dielectric response should occur at zero bias. The observed finite bias peaks are therefore suggestive of an internal electric field that breaks the degeneracy of the double‐well potential. The obvious candidate is a static intrinsic field *E*_*UC*_ that arises from a vertical gradient of the oxygen content. This built‐in field lifts the degeneracy of the double‐well potential and thus blocks the motion of the cationic dipoles, unless an antiparallel, external electric field is applied that restores the symmetry of the double‐well potential and thus eventually maximizes the cationic dielectric response. This effect is also clearly seen in the *C*_*P*_(*V*_*DC*_) loop measured for the asymmetric contact geometry in Figure 5d from a large asymmetry between the positive and negative field branches and a pronounced maximum at a finite DC voltage for only one of them.

Finally, to rule out any possibility of a crystallographic symmetry change around *T_mag_*, we have performed low‐temperature x‐ray diffraction and reciprocal space mapping to calculate in and out‐of‐plane lattice parameters (Figure S15). The results indicate no sharp change of lattice parameters at or below *T_mag_*.

## 5 Conclusions

We have measured the dielectric and magnetic properties of Nd‐HEO thin films and have shown that they are quite versatile and characteristic of a competitive magneto‐dielectric coupling. The main features of the dielectric response in the paramagnetic state at *T* ≫ *T_mag_* have been explained in terms of a phenomenological model which accounts for our main findings that: (i) The dielectric response is rather strong and composed of distinct contributions from the anionic and cationic sublattices, (ii) the polarizability of the oxygen sublattice is strongly enhanced by oxygen vacancies, (iii) a vertical gradient of the oxygen vacancies gives rise to an internal uncompensated electric field that is parallel to the surface normal, (iv) a strongly asymmetric non‐linear response arises from a clamping of the oxygen sublattice to the Nb:STO substrate, (v) the cationic response is governed by the off‐center displacement of Mn^4+^ ions that are frozen by *E_UC_*, but can be activated by applying an antiparallel external field, and finally, (vi) the dielectric response is strongly suppressed by the static magnetic order below *T_mag_* due to magnetostriction effects. Following the above discussion, Nd‐HEO emerges as a unique material, where the responses of the cationic and anionic subsystems can be decoupled by tuning the applied electric field and temperature. A simple experimental technique to investigate such 2‐component systems has also been presented in Section 2.5.

Most of the earlier work on the low‐frequency dielectric behavior in high‐entropy oxides had been performed on polycrystalline pellets, where it is difficult to distinguish the intrinsic material contribution from the structural/morphological contribution. This investigation, being performed on epitaxial thin films, adds an important piece of evidence in favor of the positive role of entropy on the enhancement of the intrinsic dielectric properties. It also provides experimental evidence of the reorganization of the oxygen vacancies, which have been theoretically postulated to stabilize a high‐entropy lattice [80]. Our work should motivate further studies of these magnetic HEO materials, for example, to fine‐tune the vertical gradient of the oxygen vacancies and the related internal field *E_UC_*, as to enhance the dielectric constant or vary the position of the side‐peaks of its electric‐field loops. The latter feature will allow one to strongly vary the nonlinearity of the dielectric response. The strength of the magneto‐dielectric coupling is another interesting parameter that can be varied, for example, by a substitution of some of the magnetic transition metal ions with non‐magnetic ones, or by a partial substitution on the A‐site of the Nd^3+^ ions by Sr^2+^ or Ca^2+^ ions which alters the hole doping level. From a fundamental science perspective, these magnetic HEO materials provide interesting possibilities for the study of competing electronic and magnetic orders in the presence of a very strong structural and electronic disorder.

A complete picture emergent from the above studies would enable the production of new generation memristors, energy storage devices, biosensors, and other magneto‐electronic devices where temperature, electric, and magnetic fields can be used to tune their dielectric constant and enhance functionality of the devices. Because of its tunability, high resistance, large dielectric constant, and high dielectric strength, Nd‐HEO is a favorable material for thin film capacitive applications. Furthermore, since there is no hysteresis in the electric field sweep above *T_mag_*, Nd‐HEO is a promising candidate for higher speed applications as compared to devices made with ferroelectric materials, which require full field sweeps to tune. Finally, since Nd‐HEO can be epitaxially lattice matched to several other perovskite materials such as manganites and high‐Tc cuprates, it can play an important role in the developing field of perovskite electronics [102] that exploits the growth of epitaxial heterostructures, and pave the way for next‐generation electronics beyond the conventional Si‐based ecosystem.

## 6 Methods

### 6.1 STEM‐EELS

Scanning transmission electron microscopy (STEM) and electron energy‐loss spectroscopy (EELS) measurements were performed using a JEOL ARM200cF operated at 200 keV, equipped with a Gatan Quantum EELS system and a spherical aberration corrector. STEM‐EELS measurements are carried out at ICTS ELECMI (CNME node, Madrid). Quantification of compositional profiles was carried out using the protocols available within the Gatan Digital Micrograph Suite.

### 6.2 Low Temperature X‐Ray Diffraction

The temperature dependence of the lattice constants of the Nd‐HEO films was measured in the ID‐01 beamline at ESRF.

### 6.3 Low‐Energy Muon Spin Rotation (LEM)

The magnetic order was investigated with muon spectroscopy at the LEM spectrometer at the muE4 beamline [103] at the Paul Scherrer Institute, Switzerland. The measurements were performed with an implantation energy of the incoming, fully spin‐polarized muons of 4 keV in a weak transverse magnetic field (100 G) geometry for temperatures ranging from 5 to 300K.

### 6.4 X‐Ray Absorption Spectroscopy and Fitting of Data Using CTM‐4XAS

X‐ray Absorption Spectroscopy (XAS) measurements were performed at the I10 beamline from Diamond Light Source, U.K. The sample was measured in total electron yield mode, at the required energy edges of the transition metals, using σ‐ and π‐polarization at 10 K and a 30° incidence angle, in ultrahigh vacuum. The XAS data of the 3d transition metal ions were simulated using CTM‐4XAS, which is a program based on crystal field theory, atomic multiplet theory, and charge transfer theory [104]. Further details are given in Section S13.

## Author Contributions

The samples were grown by R.C. and S.S. XRD and RSM at room temperature were performed in the University of Fribourg by S.S., R.C., and R.T. and were reconfirmed in the University of Geneva by S.G. STEM‐EELS characterization was performed in the Universidad Complutense de Madrid by N.B. and M.V. Dielectric and transport measurements were performed in the University of Geneva by R.C., S.S., R.T., S.G., and C. W. R. Photolithographic contact pads were fabricated by S.G. at the University of Geneva. Magnetization measurements on STO/Nd‐HEO were performed at the University of Geneva by R.C. and S.S., with the help of M.B. Magnetization measurements on LAO/Nd‐HEO were performed by S.B. at Ames National Laboratory. Muon spin rotation spectroscopy was performed at the Swiss Muon Source SµS, Paul Scherrer Institute by Z.S., R.C., S.S., and R.T. with the help of T.P. and A.S. XAS measurements were performed at Diamond Light Source by R.C., S.S., R.T., M.C., with the help of P.B. Theoretical modelling was developed by Y.P., S.O., P.M., and C.B. FIR‐Ellipsometry measurements were performed by P.M. The manuscript was prepared by S.S., C.B., Y.P., R.T., and R.C. with suggestions from M.C., P.M., N.B., M.V., S.G., Z.S. and T.P. Temperature dependence of RSM of Nd‐HEO was measured in ID‐01 beamline at ESRF with the participation of S.S., R.T., U.O. and J. Z.

## Funding

The UniFr group acknowledges funding by the Swiss National Science Foundation (SNSF) through grant Nr. 200021_212050. Yu. G. Pashkevich acknowledges the financial support of the SNSF through the individual grants IZSEZ0_212006 and IZSEZ0_215824 of the «Scholars at Risk» Program. R.C acknowledges the support by a grant of the Ministry of Research, Innovation and Digitization, CNCS‐UEFISCDI, project number PN‐III‐P1‐1.1‐PD‐2021‐0238, within PNCDI III and a grant of the European Commission within the framework of the Romanian National Recovery and Resilience Plan (PNRR) through the ESCARGOT project entitled “Enhanced Single Crystal Applications and Research in the Growth of new Optical rare earth‐based compounds for sustainable and efficient Technologies” (No: 760080/23 May 2023). Funding from the Spanish Agencia Estatal de Investigación via grant PID2021‐122980OB‐C51 is acknowledged as well. S.L.B. was supported by the U.S. Department of Energy, Office of Basic Energy Science, Division of Materials Sciences and Engineering under Contract No. DE‐AC02‐07CH11358. Part of this work is based on experiments performed at the Swiss Muon Source SµS, Paul Scherrer Institute, Villigen, Switzerland.

## Conflicts of Interest

The authors declare no conflicts of interest.

## Supporting information




**Supporting File**: adma72992‐sup‐0001‐SuppMat.pdf.

## Data Availability

The data that support the findings of this study are available from the corresponding author upon reasonable request.
