# Supplementary material for: Versatile Magneto‐Dielectric Response of Epitaxial Thin Films of the High Entropy Oxide Perovskite Nd(Cr0.2Mn0.2Fe0.2Co0.2Ni0.2)O3
Source: Adv Mater. 2026 Apr 5;38(26):e72992. doi: 10.1002/adma.72992 (PMC13155305; doi:10.1002/adma.72992)
Supplement: Supplementary file 1 — Supporting File: adma72992‐sup‐0001‐SuppMat.pdf. [file ADMA-38-e72992-s001.pdf]

Supporting information for "Versatile magneto-dielectric response of epitaxial thin films of the high entropy oxide perovskite $Nd(Cr_{0.2}Mn_{0.2}Fe_{0.2}Co_{0.2}Ni_{0.2})O_3$"

Roxana Capu, Ryan Thompson, C. Willem Rischau, Marli R. Cantarino, Premysl Marsik, Sergey L. Bud'ko, Neven Biškup, María Varela, Yurii G. Pashkevich, Serhii M. Orel, Thomas Prokscha, Andreas Suter, Jiangtao Zhao, Ugwumsinachi Oji, Marco Bonura, Peter Bencok, Zaher Salman, Stefano Gariglio, Christian Bernhard, Subhrangsu Sarkar**

Roxana Capu
Department of Physics, West University of Timisoara, Timisoara-300223, Romania

Ryan Thompson, Premysl Marsik, Yurii G. Pashkevich, Christian Bernhard, Subhrangsu Sarkar
Department of Physics and Fribourg Center for Nanomaterials, University of Fribourg, Fribourg-1700, Switzerland
Christian.bernhard@unifr.ch
Subhrangsu.sarkar@unifr.ch

C. Willem Rischau, Marco Bonura, Stefano Gariglio
Department of Quantum Matter Physics (DQMP), University of Geneva, Geneva -1211, Switzerland

Marli R. Cantarino, Ugwumsinachi Oji, Jiangtao Zhao
European Synchrotron Radiation Facility, F-38043 Grenoble Cedex 9, France

Sergey L. Bud'ko
Ames National Laboratory and Department of Physics and Astronomy, Iowa State University, Ames, Iowa 50011, United States of America

Neven Biškup, María Varela
Departamento de Física de Materiales and Instituto Pluridisciplinar, Universidad Complutense de Madrid, Madrid 28040, Spain

Yurii G. Pashkevich, Serhii Orel

O.O. Galkin Donetsk Institute for Physics and Engineering NAS of Ukraine, Kyiv-03028, Ukraine

Peter Bencok
Diamond Light Source, Harwell Science and Innovation Campus, Chilton, Didcot OX11 0DE, U.K.

Zaher Salman, Thomas Prokscha, Andreas Suter
PSI Center for Neutron and Muon Sciences CNM, Forschungsstrasse 111, Villigen, 5232, Switzerland.

Section 1

Spot to Spot variation of the Capacitance

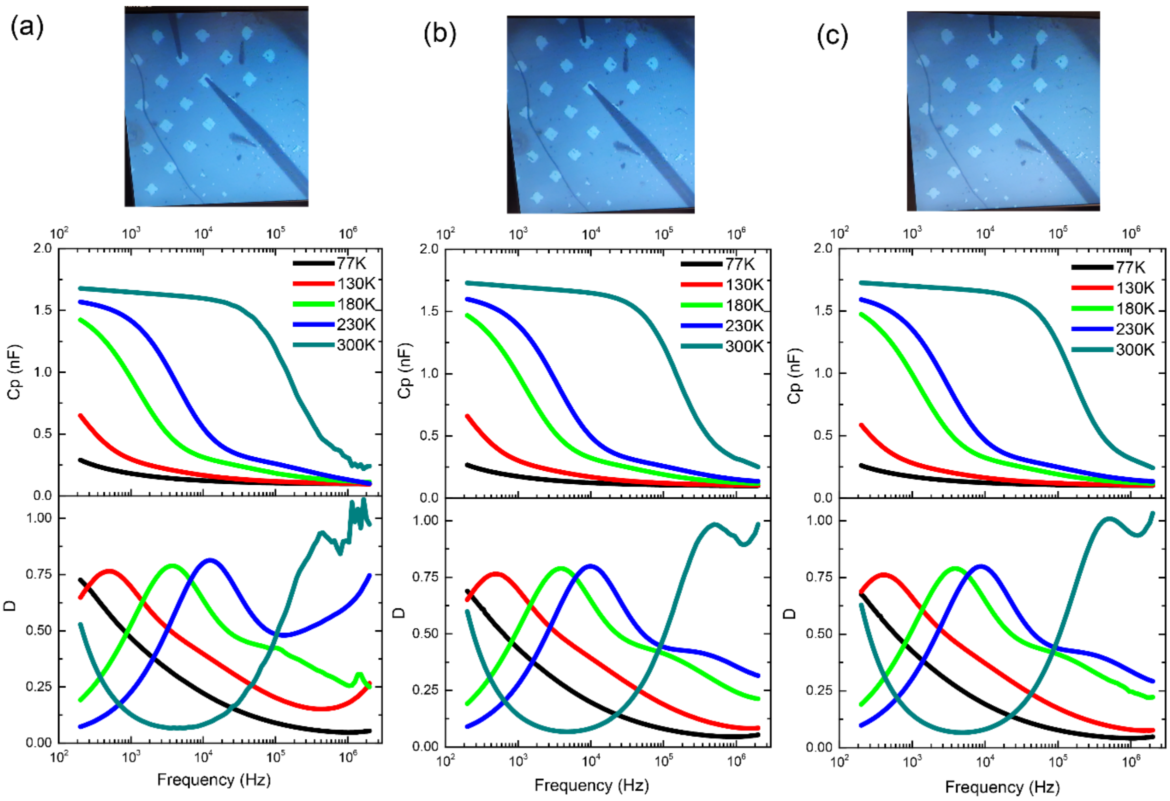

Figure S1. $C_P - D$ spectra obtained with different pairs of Pt contacts to demonstrate the homogeneity of the dielectric response of the sample.

The upper row of Figure S1 shows the probe contacts of the probe station that are touching different combinations of Pt electrodes deposited on the Nd-HEO film. The lower panels show the measured capacitive response (C_P, D vs ω) for the different contact

configurations and confirm that it is quite homogeneous over the entire sample. The signal was recorded by an Agilent E4980A LCR meter.

These samples have been investigated over more than 2 years over hundreds of field and tens of thermal cycles. During the course of measurements, the produced data remained reproducible. The zero-bias capacitance showed a reduction of 0.02 nF over 1.73 nF in the same spot during this time. The rest of the behaviour was also unchanged.

Section 2

Measurement of DC resistance

Figure S2 shows the measured I-V curves up to ± 500 mV and the resistance, R_S, that has been derived from the rather narrow region of linear response (that decreases towards low temperature). The left panel reveals an exponential increase of Rs towards low temperature (with 100 GΩ at 140 K).

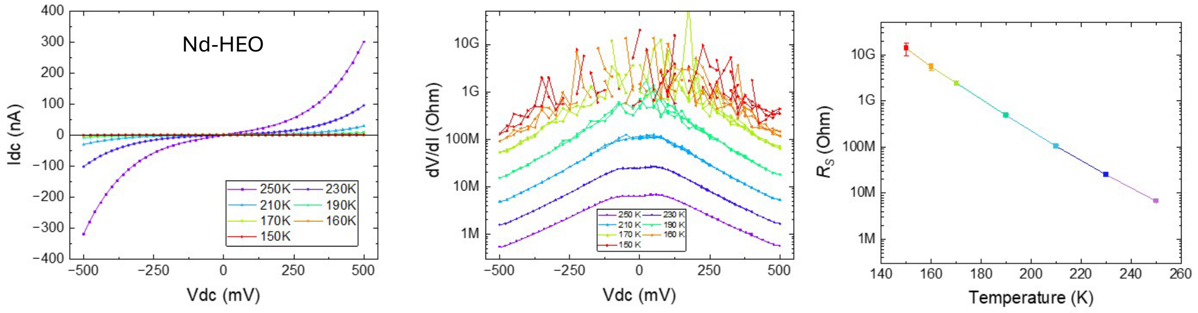

Figure S2. I-V curves, the derived dV/dI, and R_S curves of the Nd-HEO layer.

Section 3

Estimation of R_P, D from $\varepsilon''(\omega)$ using Havriliak-Negami model:[1]

Figure S3 compares the calculated parallel resistance, R_P, and dissipation factor, D, (solid lines) with the experimentally measured values of R_P and D (symbols). The imaginary part of the dielectric constant, ε_r'', has been calculated here using the parameters obtained from the best fit with the H-N model to the real part of the measured dielectric function ε_r', as shown in Figure 3.d of the manuscript. Equation S1 represents the formula of the effective resistance that takes into consideration the dc resistivity, R_{DC} (dV/dI at $V = 0$, Figure S2) and the imaginary part of the capacitance C_P'' (multiplied by a geometric factor $^{2d}/_{A}$), which act in parallel. Figure S3 (left panel) shows good applicability of the model since the simulated and measured data are quite similar. Using the formula for parallel resistors,

$$R_P = \frac{R_{DC}\left(\frac{2d}{A}\right)C_P''}{R_{DC} + \left(\frac{2d}{A}\right)C_P''} \qquad (S1),$$

where

$$C_P'' = \left(\frac{1}{2\pi f \epsilon_0 \left(\frac{2d}{A}\right) \epsilon''} \right) \tag{S2}$$

can be estimated from the imaginary part of the expression for the ac conductivity

$$Im[\tilde{\sigma}(\omega)] = Im[-j\omega\epsilon_0(\epsilon - 1)] = Im[-j\omega\epsilon_0\{(\epsilon' - 1) + j\epsilon''\}] = \omega\epsilon_0\epsilon'' \quad (S3).$$

Next, in order to calculate the dissipation factor D, Equation S4 uses the previously calculated R_P and the measured C_P. Therefore, in Figure S3 (right panel), for the different temperatures, we observe a reasonably good match between the curves of the same color, indicating parasitic contributions are sufficiently small.

$$D = \frac{1}{2\pi f C_p R_P} \tag{S4}$$

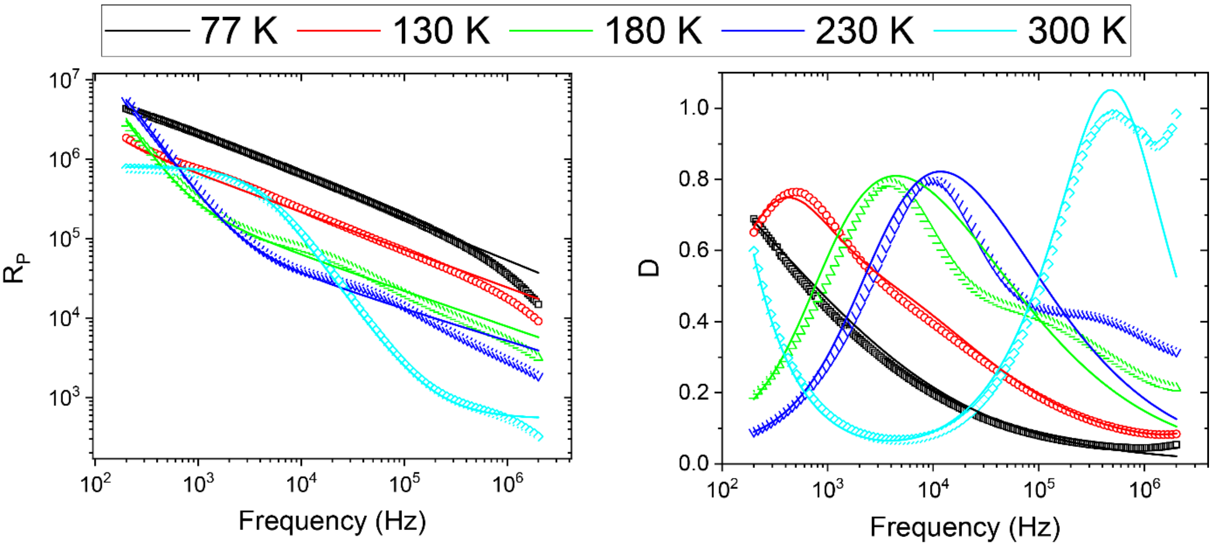

Figure S3: Comparison of the measured values of R_P and D (symbols) with those calculated using the parameters from the best fit with the H-N model (solid lines).

Section 4:

<u>$C_P - D$ and $C_P - R_P$ mode measurements of a R-C parallel circuit with standard resistors and a 1.5 nF paper capacitor:</u>

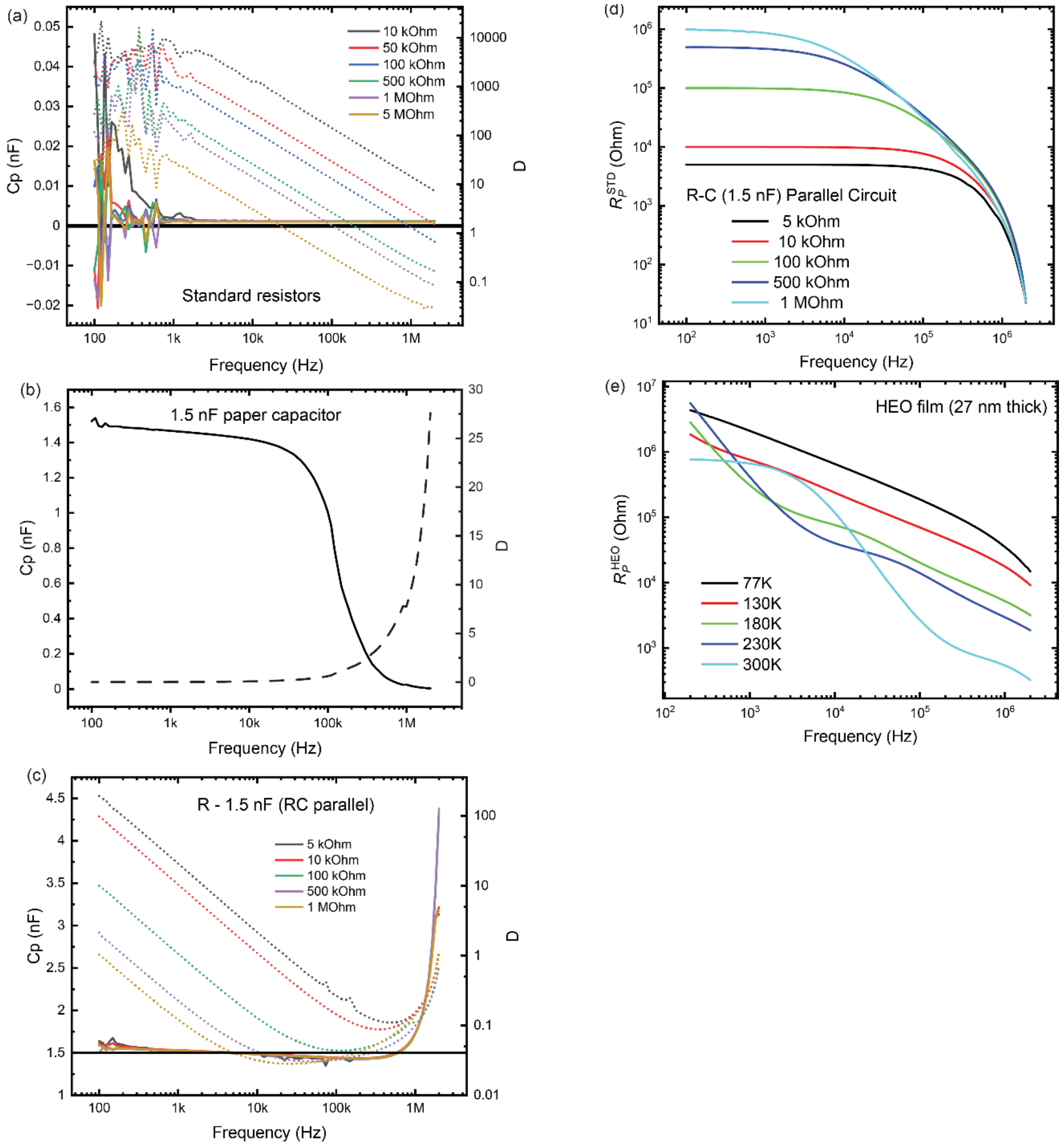

Figure S4: Frequency dependence of the capacitance and resistance of an RC parallel circuit made from various standard resistors and a 1.5 nF non-polar capacitor measured in $C_P - D$ and $C_P - R_P$ modes.

Section 5:

Temperature dependence of the finite bias peaks of the $C_P - V_{DC}$ loops:

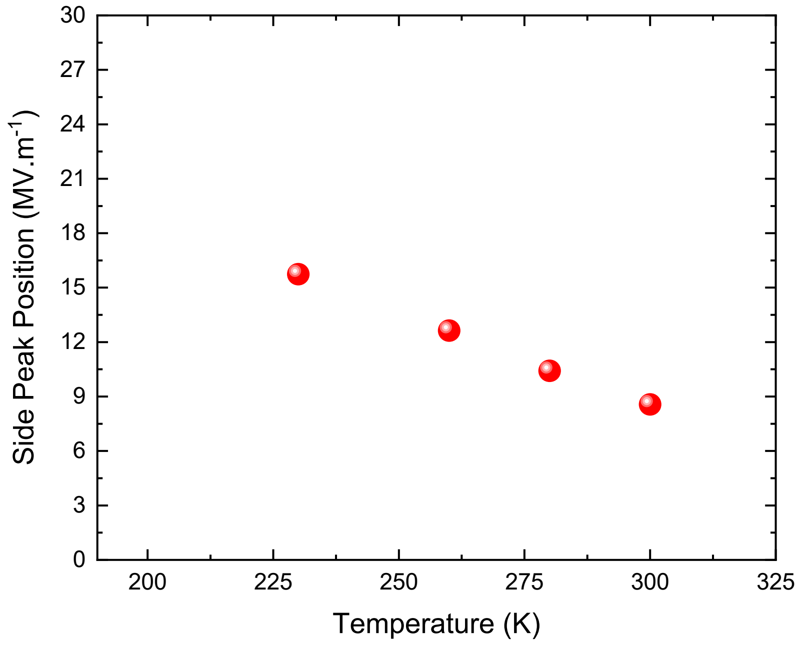

Figure S5. Temperature dependence of the position of the finite bias peaks in the C_P (V_{DC}) curves. Below 200 K their position cannot be determined anymore since their intensity is strongly reduced and their maxima are shifted outside the measured voltage range.

Section 6

Magnetization measurements for HEO grown on $LaAlO_3$

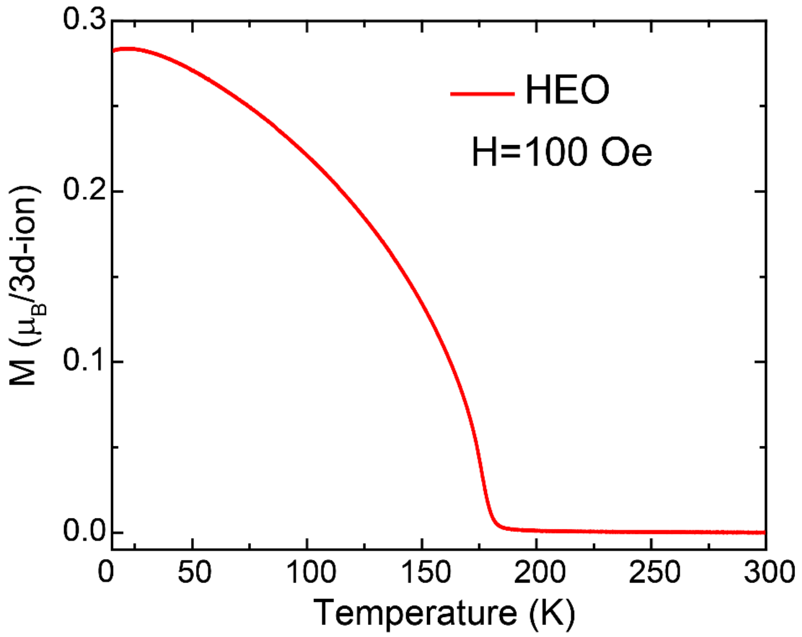

Figure S6. Temperature dependence of the dc magnetization at 100 Oe field cooling of a Nd-HEO film deposited on a $LaAlO_3$ substrate.

The magnetization measurements were performed using a Quantum Design Magnetic Property Measurement System (MPMS3) in dc mode. The sample was glued to a semi-

cylindrical quartz sample holder with a small amount of GE 7031 varnish. The applied magnetic field was parallel to the film. The measurements were done in field-cooled mode.

Section 7:

Infrared optical response of Nd-HEO film

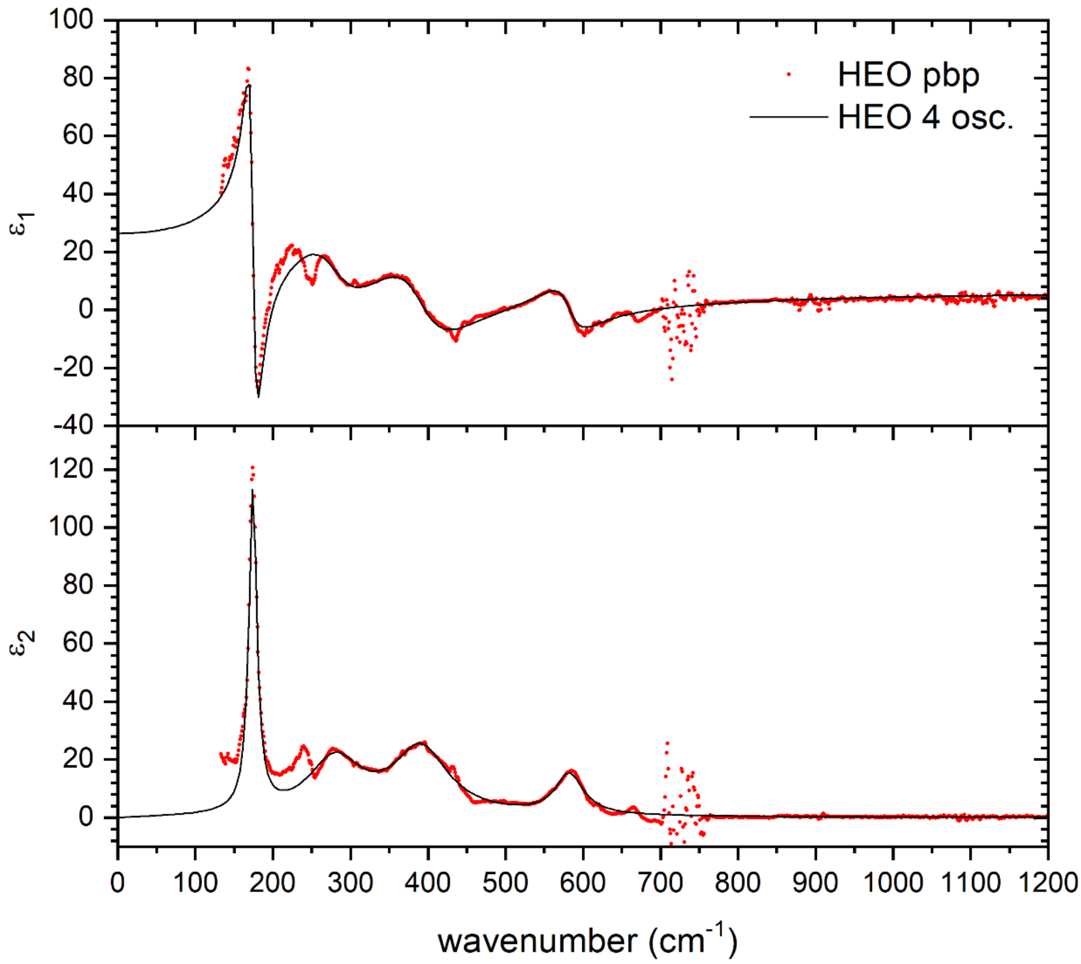

Figure S7: FIR ellipsometry analysis indicating the real and imaginary part of the dielectric constant at optical frequency range

The far-infrared dielectric response has been determined using spectroscopic ellipsometry. A 53 nm thick film of Nd-HEO grown on $YAlO_3$ (001) substrate was measured in the range 50 to 1200 cm-1 at the angle of incidence, ϕ = 75 deg.

The obtained ellipsometric data of the Nd-HEO/$YAlO_3$ sample has been analysed using a ambient/film/substrate optical model. The optical response of the orthorhombic $YAlO_3$ was known a-priori [2]. While the substrate is represented by accurate orthorhombic model, the film itself is treated as isotropic, with the film thickness fixed to value of 53 nm obtained from x-ray reflectivity experiment.

We found that the thin film response can be described sufficiently by 4 Lorentzians within Harmonic oscillator approximation:

$$\tilde{\varepsilon}(\omega)=\varepsilon_{\infty}+\sum_{j=1}^{4}\frac{\Delta\varepsilon_j\omega_{0j}^2}{\omega_{0j}^2-\omega^2-i\omega\gamma_j},$$

where the parameters $\Delta\varepsilon_j$, ω_{0j}, γ_j account for the dielectric strength, the transversal optical resonance frequency, and the damping (peak broadening), of the individual oscillators, and the ε_{∞} is the value of the dielectric constant above the phonon range. Values of the model parameters are listed in following table:

j	ω_0 (cm^{-1})	γ (cm^{-1})	$\Delta\varepsilon$
1	174	12	7.9
2	283	86	5.7
3	393	87	5.1
4	583	45	1.1
		ε_{∞}	6.6
		$\varepsilon(\omega\rightarrow0)$	26.4

Table S1. Values of the model parameters

Validity of the parameterized model was subsequently checked with an unconstrained (point-by-point) model of the dielectric response of the film fitted to the experimental data. The results are shown above on Figure S6. The discrepancies between the parameterized and unconstrained models coincide with the TO phonon mode positions of the substrate, where the sensitivity to the thin film is vanishing [3]. Particularly, the features at 240 cm^{-1} and 256 cm^{-1} coincide with strong phonon mode of the *a*-axis and *b*-axis of the $YAlO_3$ response, respectively.

Notably, the low-frequency extrapolation of the real part of the dielectric function (ε') leads to value of 26.4, far lower than the values (~254) revealed by the ac (<10^5 Hz) capacitance measurements.

Temperature dependence FIR experiments resulted in rather low-quality data (not shown) due to strong response of the substrate; however, we can still exclude any soft phonon mode phenomena in the far-infrared range. Further studies of the optical response of the high entropy oxides will be presented elsewhere.

Section 8

Example of fits of $C_P(V_{DC})$ at T= 190 K and T= 130 K using equation (7) to find out the value of $\chi_{\pm}$

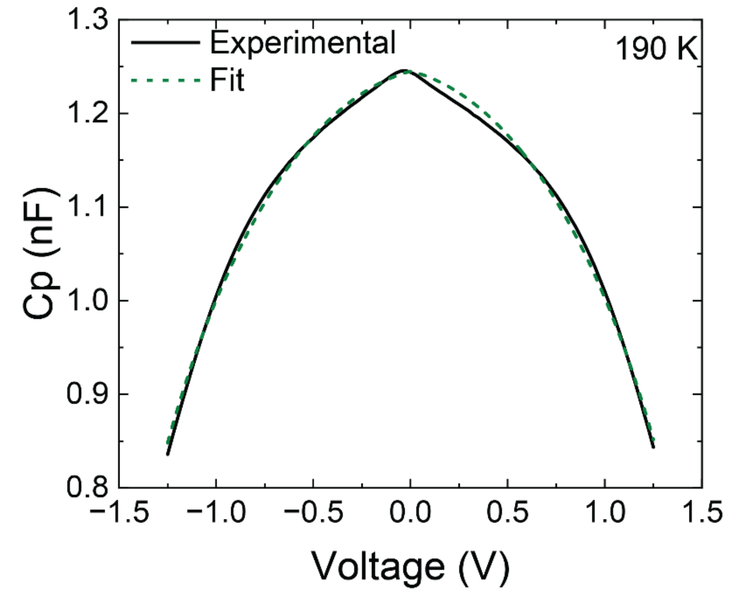

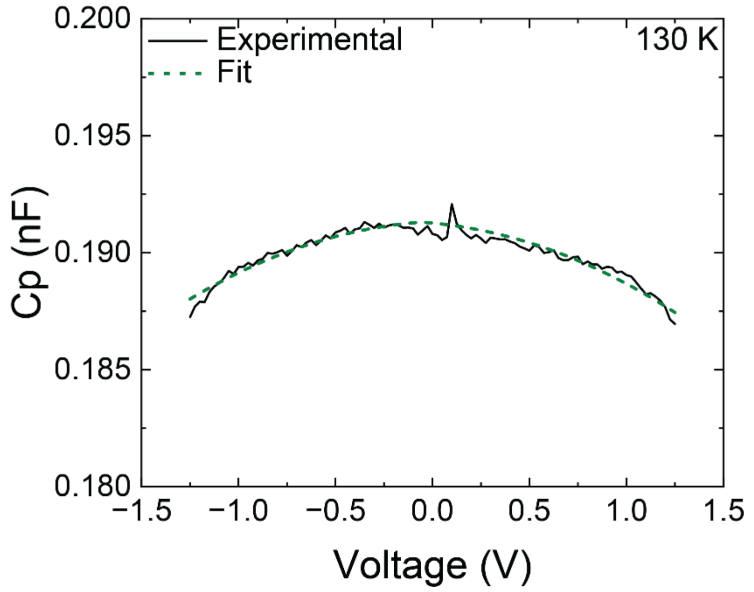

Figure S8. Demonstration of fits of $C_p(V_{dc})$ at 190 K and 130 K.

Section 9

The temperature and voltage dependence of $\varepsilon\ (T,V)$

From

$$E_1 = \frac{-\varepsilon + \chi_- E + \sqrt{(\varepsilon - \chi_- E)(\varepsilon + \chi_+ E)}}{\chi_+ + \chi_-}; \quad E_2 = \frac{\varepsilon + \chi_+ E - \sqrt{(\varepsilon - \chi_- E)(\varepsilon + \chi_+ E)}}{\chi_+ + \chi_-};$$

$$\varepsilon_1(T,E_1) = \varepsilon(T,0) + \chi_+(T)E_1; \quad \varepsilon_2(T,E_2) = \varepsilon(T,0) - \chi_-(T)E_2;$$

One can write:

$$\varepsilon_1(T,V_{DC}) = \varepsilon(T,0) + \chi_+(T)\frac{-\varepsilon + 37 * \chi_- * V_{DC} + \sqrt{(\varepsilon - 37 * \chi_- V_{DC})(\varepsilon + 37 * \chi_+ V_{DC})}}{\chi_+ + \chi_-} \quad \text{(S5)}$$

$$\varepsilon_2(T,V_{DC}) = \varepsilon(T,0) - \chi_-(T)\frac{\varepsilon + 37 * \chi_+ * V_{DC} - \sqrt{(\varepsilon - 37 * \chi_- V_{DC})(\varepsilon + 37 * \chi_+ V_{DC})}}{\chi_+ + \chi_-} \quad \text{(S6)}$$

Here $k= d^{-1}$=37x10^6m^{-1} and the notations in the above equations are the same as in the manuscript.

Section 10

A. Role of Oxygen vacancy in the apical Wycoff position

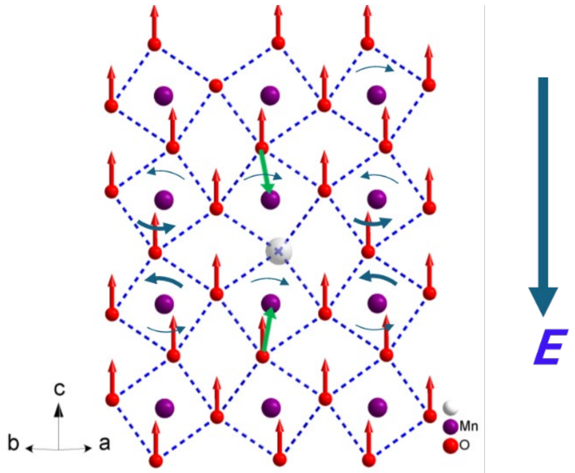

Figure S9: The oxygen vacancy at the apical Wycoff position does not contribute to further tilting of the octahedra. The cations, oxygen ions and vacancies are marked with purple, red and grey spheres.

For an oxygen vacancy at the apical position, the electric force from external field do not change the tilting angle of octahedra. The field tries to induce in-phase rotation of the two

neighbour octahedra containing a mutual vacancy. However, it is impossible due to out-of-phase rotations of the next-nearest-neighbour octahedra (the cancelling/ blocking of rotation shown by bold crooked arrows). Accordingly, the local anion density does not change.
The z – components of the huge polar moments M^{3+}–O^{2-} (green arrows) cancel each other. Hence, the regions around vacancies at the apical Wyckoff positions do not contribute to anion network flexibility and thereby dielectric contribution.

B. Estimation of the value of flexoelectric field using measurements on asymmetric circuit shown in Figure 5(d)

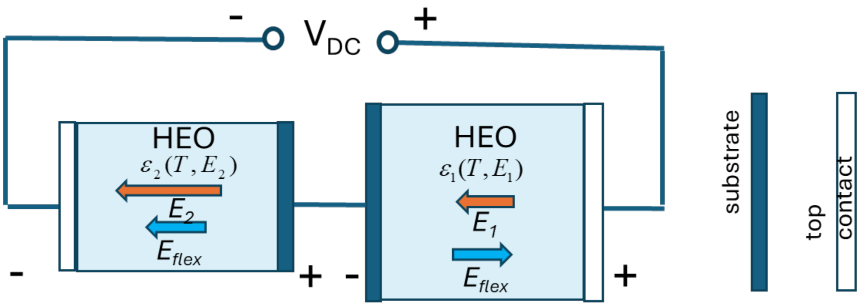

Figure S10. The sketch of asymmetric circuit used in our experiment for the case of positive bias applied on the large-pad capacitor.

For the case pad-size asymmetry denoted as *r*, and considering only anion contributions, we have relations for the field strength, capacitors, voltages and dielectric constants:

$$S_2 = S_1/r; \quad r > 1\,; \quad S_1 > S_2; \quad C_1V_1 = C_2V_2; \quad C_1 > C_2; \quad V_1 < V_2; \quad E_1 < E_2;$$

$$r\varepsilon_1E_1 = \varepsilon_2E_2; \qquad E = E_1 + E_2 = V_{DC}/d$$

$$\varepsilon_1(T,E_1) = \varepsilon(T,0) + \chi_+(T)E_1; \quad \varepsilon_2(T,E_2) = \varepsilon(T,0) - \chi_-(T)E_2; \quad \varepsilon(T,0) = \varepsilon(T) = \varepsilon;$$

Dependencies of the field strength on applied voltage:

$$E_1 = \frac{-\varepsilon(r-1) - 2(\varepsilon - \chi_-E) + \sqrt{\varepsilon^2(r-1)^2 + 4r(\varepsilon - \chi_-E)(\varepsilon + \chi_+E)}}{2(r\chi_+ + \chi_-)} \qquad (S7)$$

$$E_2 = \frac{\varepsilon(r-1) + 2(\varepsilon + r\chi_+E) - \sqrt{\varepsilon^2(r-1)^2 + 4r(\varepsilon - \chi_-E)(\varepsilon + \chi_+E)}}{2(r\chi_+ + \chi_-)} \qquad (S8)$$

The respective dependencies for the case r= 16 and at room temperature with ε=274 and under assumption that χ_-=-χ_+= 2m/MV are shown in Figure S11. It is accounting for that maximal bias 1.5 V corresponds to we maximal field strength E=55.5 MV/m.

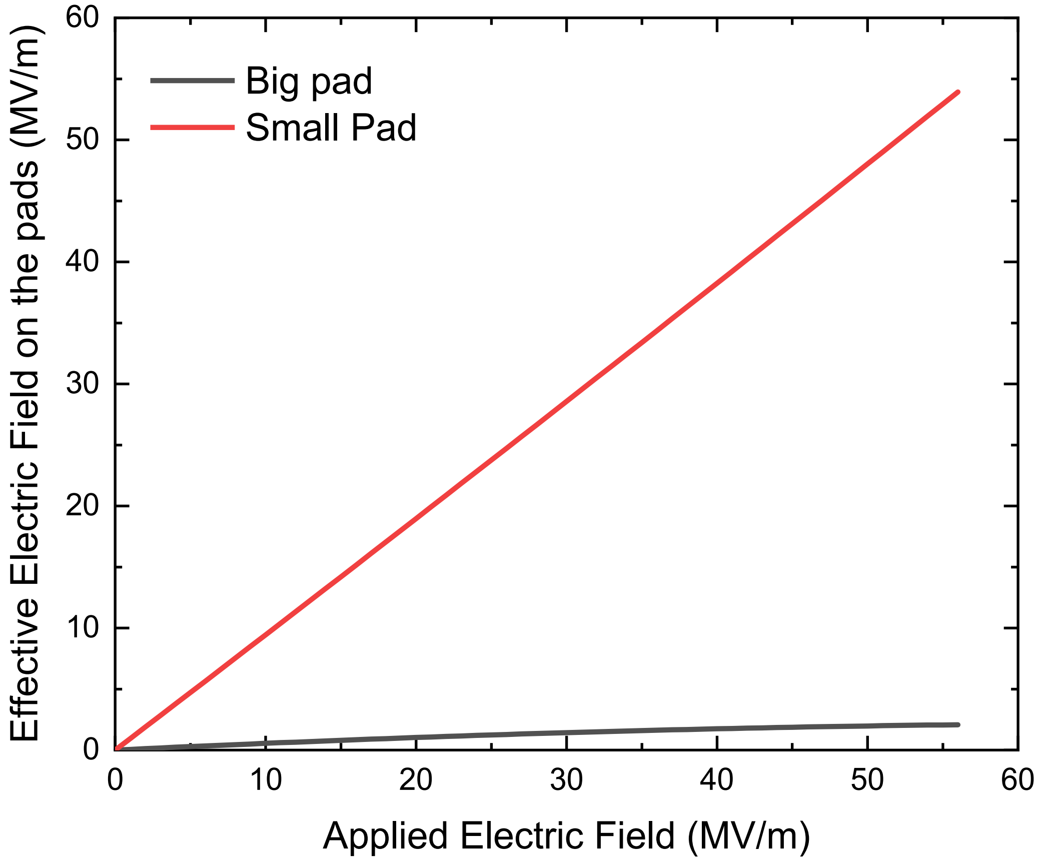

Figure S11. The change in electric field observed by the big and small pad as a function of applied electric field for the circuit corresponding to Figure 5(d) in the main manuscript.

As follows from our calculations for the case of positive voltage applied on large pad capacitor the external field directed against flexoelectric field (see Figure S10). However, its maximal value ~2 $MV.m^{-1}$ is not enough to induce side peak by rearranging cations collected by flexoelectric field. From this estimation we deduce that lowest level of the flexoelectric field must be higher than 2 $MV.m^{-1}$.

Section 11

<u>Cation contribution caused by the off-centring process</u>

In this Section we will consider the details of the lattice structure of Nd-HEO; namely the mutual positions, anions and cations and distances between them.

First, we proceed with an investigation of the cation-ligand distances in the Nd-HEO lattice structure. As a starting point we use structural data for the $NdMnO_3$ lattice in *Pbnm* symmetry reported in [4]. In this setting the *c*-axis is perpendicular to the film surface, and the primitive cell contains four formula units. There we replaced initial lattice constants a=5.5183 Å, b=5.7058 Å, c=7.7725 Å by Nd-HEO lattice constants a= b= 5.5204 Å and

c=7.6140 Å (as found from the RSM in Figure 1), and suppose that the Wyckoff positions of all ions remain the same. In this 0th-order approximation, each cations are in the centre of an oxygen octahedra and the coordinates of all oxygen ions are known. The next considerations will be based on this approximation for anion network.

The off-centring of the cations is possible only if the metal-ligand distances are larger than the sum of the Shannon ionic radii [5]. The real off-centring process is feasible if the cation's electronic ground state energy decreases under the cation's displacement. The corresponding energy-displacement function is referred to as the cation's adiabatic potential. The most probable off-centring process occurs in the direction that minimizes the adiabatic potential. The off-centring stops if the cation sphere with the corresponding Shannon radius touches one, two or three anion spheres (we choose Shannon radius for each oxygen equal to 1.38 Å [5]).

Thus, as a first step to find possibility for off-centring of cations, one should consider geometries using Shannon radii for all types of cations located in an octahedral environment of ligands and each of which we consider to only possess +2, +3 and +4 oxidation states [5]. Note, that ionic radius decreases if oxidation increases [5]. Due to *Pbnm* symmetry, only one type of octahedron can be considered because all four octahedra in the unit cell are equivalent and can be obtained from each other by symmetry operations. Furthermore, the site-symmetry of cations in the centre of octahedron consists of inversion that provides full equivalence for off-centring in two opposite directions and, in turn, creates a double-well adiabatic potential with equal depths of wells.

We calculate the change of adiabatic potential along defined off-centring directions up to the known coordinate for the maximal displacement at which cation's and anion's Shannon spheres touch each other. The calculation for all types of cations has been performed using modified crystal field theory (MCFT) [6-8]. The MCFT operates with full orthonormal set of many-electron hydrogen-like wave functions for a given $3d^n$ electron configuration of the cation. The spin-orbit coupling is incorporated into MCFT. The crystal field potential formed by the known ligand's charges and ligand's positions which are the arbitrary input parameters of the MCFT. The main theoretical parameter is an effective nuclear charge Z_{eff} of the metallic ion which defines the crystal-field strength and degree of covalency of the metal-ligand bonds. Due to the screening by the cloud of ligand electrons the Z_{eff} of metal ion in a crystal field environment is smaller compared to effective nuclear charge of the free ion. It is shown in ref [6] that well known Tanabe-Sugano diagrams for variables "energy-levels, crystal-field strength" can be reproduced in the variables "energy-levels, effective nuclear

charge”. Earlier, the manifestation of the adiabatic potential variation in experimental EPR spectrum of Cu^{2+} ions doped into spinel $ZnAl_2O_4$ has been investigated with help of MCFT in ref [7].

We observe that off-centring for cations with 2+ oxidation state is impossible due to simple geometrical reasons (with particular attention to Ni^{2+} and Co^{2+} ions which were observed in XAS experiment). A similar analysis shows that cations with the +3 valence state have no ability for off-centring in the above-mentioned approximate description of the regular anionic network. Moreover, in Shannon's touching sphere model, practically all cations in the +3 valence state cannot be accommodated in the anion’s octahedra. One can suppose that under growth process this is a main reason for the observed gradient of the oxygen density.

The same geometrical analysis has been done for all M^{4+} cations. It was shown that off-centring can be realized only for Mn^{4+}, Co^{4+} and Ni^{4+} ions. Next, we focus on the possible off-centring of the Mn^{4+} ion (Shannon radius 0.54 Å [5]), since the latter two ions were not observed in our XAS measurement.

The MCFT calculations combined with geometry considerations reveal two distinctive directions for Mn^{4+} off-centring as they are shown in Figure S12. For both directions the stop-point vectors (i.e. maximum off-centring displacements) are:

$$\boldsymbol{R}_{\mathrm{I}} = \pm [-0.00044, 0.1595, 0.0534]\ \text{Å};$$

$$\boldsymbol{R}_{\mathrm{II}} = \pm [-0.14987, -0.0701, 0.0196]\ \text{Å}; \qquad \text{(S9)}$$

Notice, the maximum off-centering displacements for Mn^{4+} ion position in other three octahedra in the unite cell can be obtained by 2x, 2y, 2z rotation of the vectors in Eq.S1.

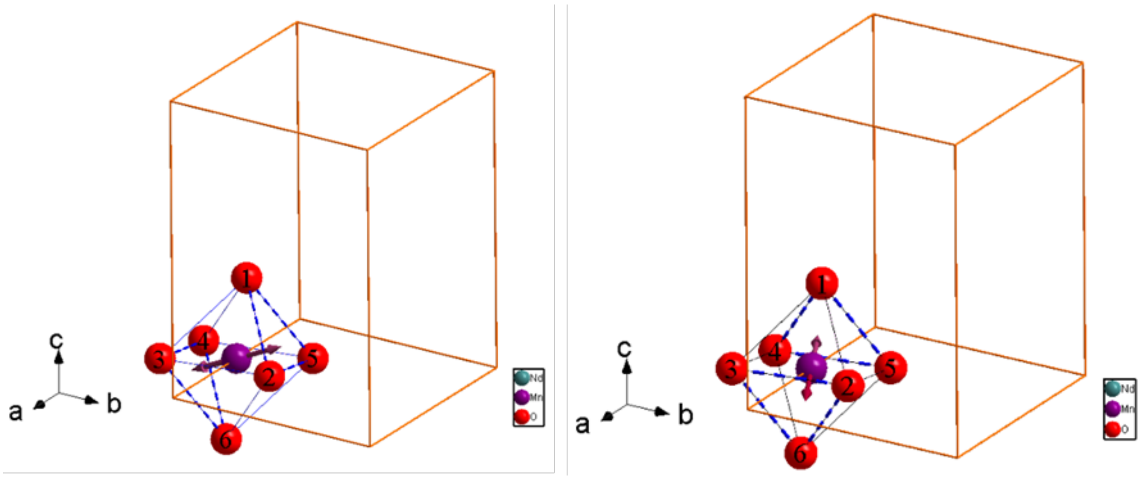

Figure S12. Two types of off-centring directions for Mn4+ ions in the oxygen octahedron cage (shown by arrows). The equivalent displacements: between [1;2;5] and [3;4;6] oxygen triangles (case I - left side); in between [1;4;5] and [2;3;6] oxygen triangles (case II-right

side). The oxygen triangles with respective enumeration of oxygen ions are marked by dashed blue lines.

The result of MCFT calculations for the change of Mn^{4+} adiabatic potential along displacement directions $\boldsymbol{R_I}$ and $\boldsymbol{R_{II}}$ is shown in Figure S13.

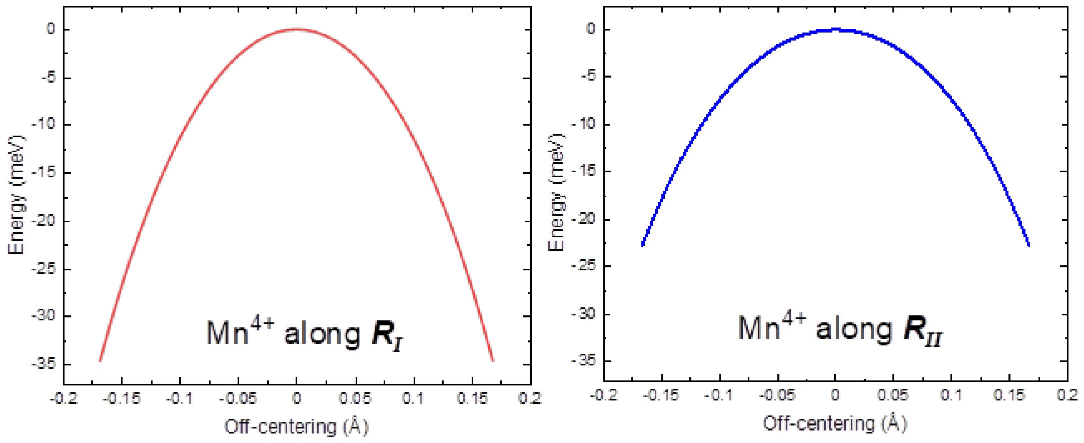

Figure S13. The variation of the Mn4+ adiabatic potential under off-centering displacement for two types directions, along: RI -left side, and along RII -right side.

The barriers between wells in the calculated double-well adiabatic potentials for case I and case II of Equation S9 are 34.625 meV and 22.75 meV respectively. Under temperature decrease and under absence of the external and internal electric field, each well is equally populated.

Note, that in the real anion sublattice with various local octahedron deformation, one can expect presence of a set of double-well potentials with various barrier-heights between the wells. Next, we focus on the realization of the case I as the main representative of a set double-well potential.

The Mn^{4+} off-centering creates polar moments with maximal value $\boldsymbol{p_{I/II}}=4|e|\boldsymbol{R_{I/II}}$. Under averaging on all possible positions of Mn^{4+} ions in the four octahedra of the unite cell, the total polar moment caused by off-centring is zero. Presence of an internal/external field along the c-axis induces polar moment along the c-axis with a maximal value $p_z = 4|e|\times$ 0.0534 Å. However, the in-plane polar moments still cancel each other out.

One can estimate the maximal polarization of unite cell containing four octahedra with 1/5 probability for Mn^{4+} - population on each octahedron:

$$P = \frac{p_{cel}}{v_0} = \frac{4p_z}{5v_0} = \frac{4*4|e|0.0534A}{5v_0} = 0.0112\frac{C}{m^2};$$

where v_0 =244.7 Å^3 is the unite cell volume. The value of corresponding electric field arising from maximally polarized cation subsystem at room temperature (i.e. with ε=274) equals to:

$$E_{cat} = \frac{P}{\varepsilon_0 \varepsilon} = \frac{4\pi P}{4\pi \varepsilon_0 \varepsilon} = 4.62 \frac{MV}{m}$$

We approximate the adiabatic potential for the case I by Morse potential as displayed in Figure 10(a) of the main manuscript.

The calculated vibration energy levels are at -24.54875 meV, -9.58425 meV and -1.53025 meV. The approximation is based on the known value of the $NdMnO_3$ polar mode with energy 21.875 meV with polarization along the *b*-axis. Namely this mode is relevant to the observed off-centring which occurs with the maximum off-centring amplitude occurs in the ab-plane along the b-axis.

Section 12

XAS Simulations

In order to understand the experimentally obtained XAS spectra, we used the program CTM4XAS[9] to simulate the $L_{2,3}$ edges of Ni, Fe, Cr, Mn and Co. CTM4XAS is a semi-empirical simulation software that explicitly includes important interactions necessary to calculate the spectra of L edges which cannot be calculated using DFT-based codes. Primarily, this includes the core and valence spin-orbit coupling, the core-valence overlap (i.e. multiplet effects), and the effect of strong correlations within the charge transfer model.

As CTM4XAS is simulation software and not fitting software, the parameters must be carefully chosen in order to reproduce the experimental data. Starting parameters were chosen following the work of [10], which studied $Dy(Fe_{0.2}Mn_{0.2}Co_{0.2}Cr_{0.2}Ni_{0.2})O_3$, a similar compound to Nd-HEO, which replaces Nd with Dy in the A-site. These parameters were then modified to best fit our spectra. A summary of the parameters used in this work can be found in Table S1.

In summary, we find that Ni is in the +2 oxidation state, Mn is in the +4, Fe and Cr are fully +3, and Co has a nearly 50/50 mixture of +2 and +3 oxidation states.

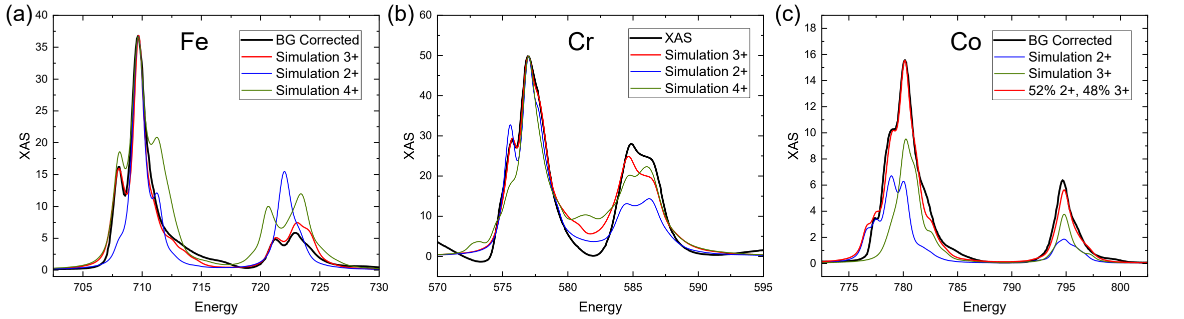

Figure S14. Background corrected X-ray absorption spectra (black line) and simulated spectra for 2+ (blue lines), 3+ (red lines) and 4+ (green lines) valency states of L2,3 edges of (a) Fe, (b) Cr, and (c) Co.

**Parameter**	**Ni**	**Mn**	**Fe**	**Co**	**Cr**
Symmetry	Oh	Oh	Oh	Oh	Oh
Oxidation State	+2	+4	+3	52% (+2) +48% (+3)	+3
10Dq	2.2	2.1	1.7	2+: 2.0 eV 3+: 0.6 eV	1.5
Charge Transfer Δ	-	2	-	-	0
Fdd	80%	32%	56%	2+: 70% 3+: 60%	80%
Fpd	80%	56%	80%	2+: 70% 3+: 60%	80%
Gpd	80%	48%	64%	2+: 70% 3+: 60%	80%
Lorentzian broadening	L3: 0.48 eV L2: 0.52 eV	L3: 0.3 eV L2: 0.45 eV	L3: 0.35 eV L2: 0.5 eV	0.38 eV (all)	L3: 0.3 eV L2: 0.6 eV
Gausssian broadening	0.2 eV	0.2 eV	0.2 eV	0.2 eV	0.2 eV
SO Coupling Reduction	1	1.05	1.02	2+: 1.02 3+: 0.97	1

Table S2. List of parameters for Ni, Mn, Fe, Co, and Cr L2,3 edges used for simulated XAS spectra shown in Figure 9 of the main text and Figure S13 of the Supporting Information.

Section 13

Temperature dependence of lattice constant of Nd-HEO

The results of in and out-of-plane lattice constant was calculated from synchrotron-based reciprocal space mapping at ID-01 beamline[11] in ESRF are presented in Figure S14. The in-plane lattice parameter shows an uneven temperature dependence due to the broadening of the RSM spot at low temperature, probably due to misalignment of the zone axis.

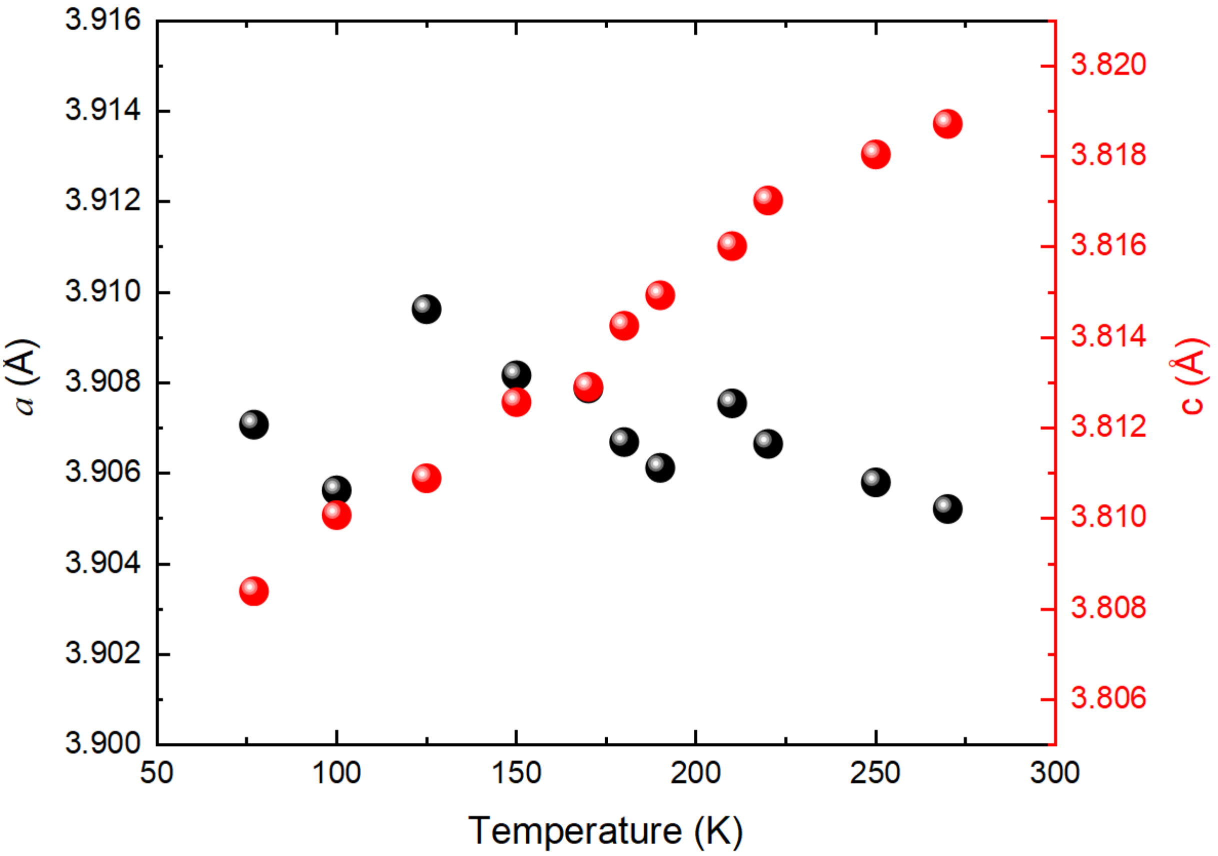

Figure S15. Temperature dependence of the in-plane (*a*) and out-of-plane (*c*) lattice constant of Nd-HEO exhibiting no change in crystallographic symmetry down to 77 K.

References:

[1] Alexei A. Bokov & Ye, Z.-G. "Dielectric relaxation in relaxor ferroelectrics. Journal of Advanced Dielectrics" **2012**, 2,1241010. Doi:https://doi.org/10.1142/S2010135X1241010X.

[2] Bugnon, L., Bernhard, C. & Marsik, P. "Broad-band ellipsometry study of the anisotropic dielectric response of $YAlO_3$" **2025**, arXiv 2403.18392. Doi:https://doi.org/10.48550/arXiv.2403.18392.

[3] Marsik P., et al. "Ellipsometry study of the infrared-active phonon modes in strained SrMnO3 thin films" Advanced Optical Technologies **2022**, 11, 179. Doi: https://doi.org/10.1515/aot-2022-0009

[4] Nandy A. & Pradhan, S. K. "Effects of monovalent cation doping on the structure, microstructure, lattice distortion and magnetic behavior of single crystalline $NdMnO_3$ compounds". Dalton Transactions **2015**, 44, 17229. Doi:https://doi.org/10.1039/C5DT02154E.

[5] Shannon, R. D. "Revised effective ionic radii and systematic studies of interatomic distances in halides and chalcogenides". Acta Cryst. **1976**, A32, 751. Doi:https://doi.org/10.1107/S0567739476001551.

[6] Lamonova K. V. et al. "Intermediate-spin state of a 3d ion in the octahedral environment and generalization of the Tanabe-Sugano diagrams" The Journal of Physical Chemistry A **2011**, 115, 13596. Doi:https://doi.org/10.1021/jp2071265.

[7] Lamonova K.V., Orel S.M. & Pashkevich, Yu. G. "Modified crystal field theory and its applications" 200 edn, (PH "Akademperiodyka", 2019). Doi: https://doi.org/10.15407/akademperiodyka.377.224

[8] Shapovalov V. A. et al. "Multi-minimum adiabatic potential in the single crystal normal spinel $ZnAl_2O_4$, doped by Cu^{2+} ions" J. Phys.: Condens. Matter **2010**, 22, 245504. Doi:https://iopscience.iop.org/article/10.1088/0953-8984/22/24/245504.

[9] Stavitski, E. & deGroot, F. M. F. "The CTM4XAS program for EELS and XAS spectral shape analysis of transition metal L edges" Micron **2010**, 41, 687. Doi:https://doi.org/10.1016/j.micron.2010.06.005.

[10] Cocconcelli M. et al. "Spin reorientation in Dy-based high-entropy oxide perovskite thin films" Phys. Rev. B **2024**, 109, 134422. Doi: https://doi.org/10.1103/PhysRevB.109.134422

[11] Chahine G. A. et al., Imaging of strain and lattice orientation by quick scanning X-ray microscopy combined with three-dimensional reciprocal space mapping, J. Appl. Cryst. **2014**, 47, 762 Doi:https://doi.org/10.1107/S1600576714004506
